# Trace-level ruthenium incorporated with transition metal components enabled the development of high-efficiency Li–CO_2_ batteries

**DOI:** 10.1039/d6sc05155c

**Published:** 2026-08-14

**Authors:** Jingchun Sun, Qingqing Jiang, Xingyu Li, Guangle Zeng, Xiaole Han, Yi Liu, Tengfei Zhou, Juncheng Hu

**Affiliations:** a Key Laboratory of Catalysis and Energy Materials Chemistry of Ministry of Education, School of Chemistry and Materials Science, South-Central Minzu University Wuhan 430074 China qqjiang@mail.scuec.edu.cn jchu@mail.scuec.edu.cn; b Institutes of Physical Science and Information Technology, Anhui University Hefei 230601 China

## Abstract

Li–CO_2_ batteries offer high energy density and carbon neutrality potential, whereas their practical applications are severely hampered by low energy efficiency arising from sluggish redox kinetics and tough mass transfer. Herein, trace-level noble metal ruthenium (Ru) could boost the performance of Li–CO_2_ batteries through hybridization of 4d orbitals with multi transition metal components (Ni, Cu, Zn, and Cd) and following electronic redistribution. The rich electronic environment and diverse metal sites could tune the adsorption energies of CO_2_ and reaction intermediates to accelerate the reaction kinetics. The NiCuZnCdRu/CF cathode displays an ultralow voltage gap (1.19 V) with a high energy efficiency of 97.2% as well as stable service life exceeding 2500 h at 20 µA cm^−2^. This study offers feasible design for durable cathodes in advanced Li–CO_2_ batteries through multi-metallic synergy which could optimize the charge transfer and enable the spatial distribution of Li_2_CO_3_.

## Abstract 2

A trace multi-metallic (Ru, Ni, Cu, Zn, and Cd) catalyst anchored on carbon fiber regulates Li_2_CO_3_ distribution and accelerates CO_2_RR/CO_2_ER kinetics, enabling Li–CO_2_ batteries with a 1.19 V voltage gap and high energy efficiency (97.2%).

## Graphical abstract

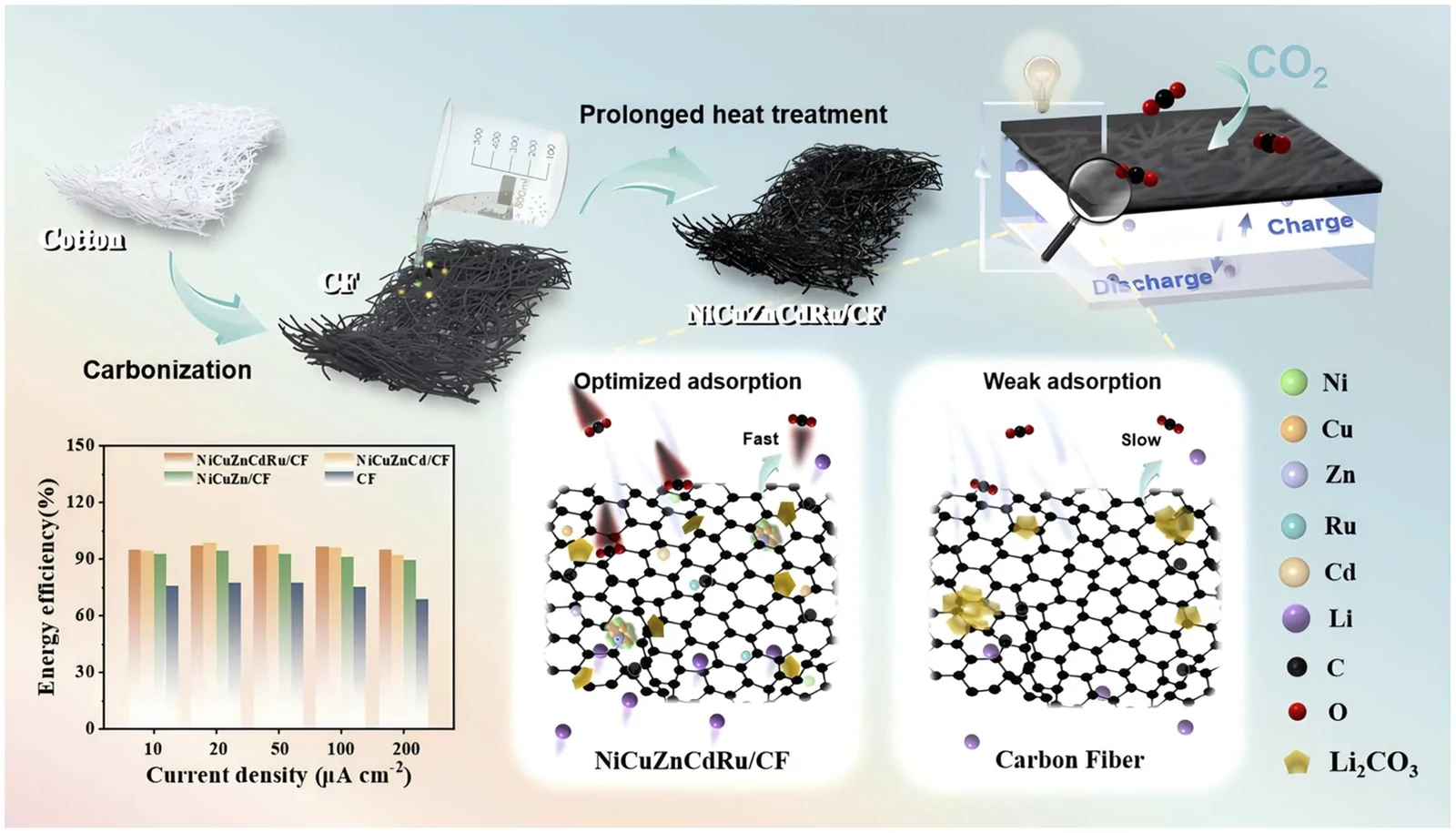

## Introduction

Lithium–carbon dioxide (Li–CO_2_) batteries have drawn considerable attention due to their dual capability of CO_2_ fixation and energy storage. Through the typical four-electron transfer reaction 4Li^+^ + 3CO_2_ ↔ 2Li_2_CO_3_ + C, Li–CO_2_ batteries could achieve a theoretical energy density of 1876 Wh kg^−1^.^1–4^ They show great application potential in CO_2_-rich scenarios such as deep-sea investigation and Mars exploration. However, the energy efficiency is usually below 80% in conventional Li–CO_2_ batteries which limits their commercial potential. This could be attributed to irreversible decomposition of Li_2_CO_3_ with extremely low electrical conductivity,^5,6^ and sluggish kinetics accompanying the formation of complex intermediates during reduction and evolution reactions (CO_2_RR/CO_2_ER).^7^ Besides, the large voltage polarization due to the unstable mass transfer across the electrochemical interface (solid–liquid–gas) should not be neglected.^8,9^ To develop practical Li–CO_2_ batteries, resolving the aforementioned issues to increase the energy efficiency is highly essential.

Various auxiliary strategies, such as photocatalysis, redox mediators, and solid-state electrolytes have been devoted to enhance the energy efficiency beyond 95%; nevertheless, they often suffer from high system complexity, poor interfacial compatibility and excessive cost.^10–12^ Consequently, the development of efficient cathode catalysts remains a crucial pathway toward practical Li–CO_2_ batteries. The conventional single-metal and bimetallic catalysts are constrained by their insufficient active site diversity for effectively facilitating the complex multi-step reactions in Li–CO_2_ batteries.^13^ Multicomponent catalytic systems with synergistic interactions have emerged as a promising direction. Chen *et al.* reported a hexa-high entropy alloy PtRuZnCoNiCu cathode which enabled an oxalate formation pathway and achieved a 3.06 V discharge voltage and 91% energy efficiency.^14^ The multicomponent systems leverage synergistic interactions to provide diverse active sites, modulate reactant adsorption and diffusion on the cathode, optimize CO_2_ reaction pathways, and ultimately promote uniform deposition and highly reversible conversion of discharge products, leading to improved overall energy efficiency and catalytic sustainability.^7,14–17^ However, it is a fact that the alloy cathode faces variable difficulties, such as complex synthesis procedure, stringent equipment requirements, and susceptibility to component segregation or nanoparticle agglomeration, which cannot meet the practical application and the need for a large three-dimensional surface in gas cathodes.

The influence of trace metal doping on battery electrochemical properties has attracted growing interest with advantages in reducing the catalyst prices but increasing element utilization efficiencies. Trace metals retained in carbon nanotubes (*e.g.* Fe, Co, and Ni) can induce electron aggregation at the metal–carbon interface to promote charge transfer during the reaction, significantly reducing the multi-step reaction barrier.^18^ These resource-rich 3d metals have been widely investigated to optimize catalytic performance, but their corrosion resistance and long-term stability under electrochemical conditions still remain inadequate.^19,20^ In comparison, the 4d orbital electrons have a broader spatial distribution, and can undergo more significant hybridization with the surrounding ligand orbitals.^21^ Similarly trace Ru can form Ru–O–Zr/Ce active sites that promote electron transfer from the C=O bond of CO_2_ to Ru species and consequently enhance CO_2_ adsorption while accelerating electrocatalytic reduction.^22^ The 4d metals provide additional orbital degrees of freedom which can be used to finely adjust the electron band structure and the adsorption and desorption energetics of reaction intermediates.^23^ These properties are of great significance for constructing stable and highly active interface structures, improving battery energy efficiency and cycle stability.

In this work, trace-level ruthenium was incorporated with transition metal components (Ni, Cu, Zn, and Cd) to modulate the fine electronic structure through hybridization of 4d orbitals. Combined with the cotton derived flexible carbon fibers as the supporting matrix, the NiCuZnCdRu/CF cathode could precisely regulate the growth path and spatial morphology of discharge products as well as effectively inhibit the local excessive accumulation of the products, thereby maintaining the efficient mass transfer at the three-phase interface. This rationally designed electrode further secures stable interfacial kinetics and charge transfer. The NiCuZnCdRu/CF cathode exhibits a narrow voltage gap of 1.19 V and a high energy efficiency of 97.2% at 20 µA cm^−2^, sustaining stable operation for over 2500 h. Even at 100 µA cm^−2^, it maintains an energy efficiency of 91.6% after 500 cycles, demonstrating its potential for long-life, high-rate Li–CO_2_ batteries.

## Experimental

### Preparation of materials

The commercial cotton was processed by washing and sizing, followed by carbonization under an argon atmosphere at 1100 °C for 2 h to form carbon fibers (CFs). A precursor solution was prepared by dissolving 0.01 mmol Ni(NO_3_)_2_·6H_2_O, Cu(NO_3_)_2_·3H_2_O, Zn(NO_3_)_2_·6H_2_O, Cd(NO_3_)_2_·4H_2_O, and RuCl_3_ in 5 mL ethanol, followed by stirring the mixture at room temperature for 6 h. The precursor solution was directly dropped onto the CF with a loading of ∼200 µL cm^−2^ and dried at 60 °C for 12 h in a vacuum oven. The annealing process was conducted in a tube furnace under a 10% H_2_/Ar atmosphere, while it was heated to 1100 °C (1 °C min^−1^) for 2 h. The NiCuZnCdRu/CF component was fabricated through a melting process and a thermodynamically driven phase transition. Contrast cathode materials were prepared by changing the composition and concentration of the precursor solution.

### Electrochemical characterization

The standard coin-type (CR2032) Li–CO_2_ batteries were assembled in a glovebox filled with argon. The battery configuration comprised a lithium foil anode, a flexible NiCuZnCdRu/CF catalyst (1 cm × 1 cm) cathode, and a glass fiber separator, and incorporated an electrolyte of 1 M LiTFSI (100 vol%) in tetraglyme. The entire assembly was housed in a custom-made sealed vessel filling with CO_2_. Battery performance was evaluated using a NEWARE testing system. Cyclic voltammetry (CV) and electrochemical impedance spectroscopy (EIS) were performed using a CHI760E electrochemical workstation. The distribution of relaxation times (DRT) was derived from the *in situ* EIS data. For *ex situ* characterization, batteries were disassembled at specific states during the charge–discharge process, and the electrodes were recovered and dried in an Ar atmosphere. The differential electrochemical mass spectrometry (DEMS) analysis was performed using an HIDEN HPR20 system. The flow was regulated using a Bronkhorst mass-flow controller. After the cell and gas-transfer lines had been purged with 10% CO_2_/Ar at 2.0 mL min^−1^ for 0.5 h, the DEMS system was connected to an *in situ* electrochemical cell and controlled by using a LAND system. The cell was charged and discharged at a constant current of 0.5 mA for 0.5 h in each process with a carrier gas flow rate of 0.5 mL min^−1^, while the CO_2_ flux was simultaneously monitored.

## Results and discussion

### Synthesis and characterization of NiCuZnCdRu/CF catalysts

The synthesis scheme of the NiCuZnCdRu/CF cathode is depicted in Fig. 1. NiCuZnCdRu nanoparticles were successfully anchored on the carbon fibers (CFs) by drop-casting a transition metal precursor solution onto the CF, followed by a simple and melting process and a thermodynamically driven phase transition. During the slow heating stage, the precursors decomposed and were reduced into metal clusters that were confined in the CF matrix. Upon reaching the pyrolysis temperature, rapid atomic diffusion enabled the formation of uniformly dispersed nanoparticles.^17^

**Fig. 1:**
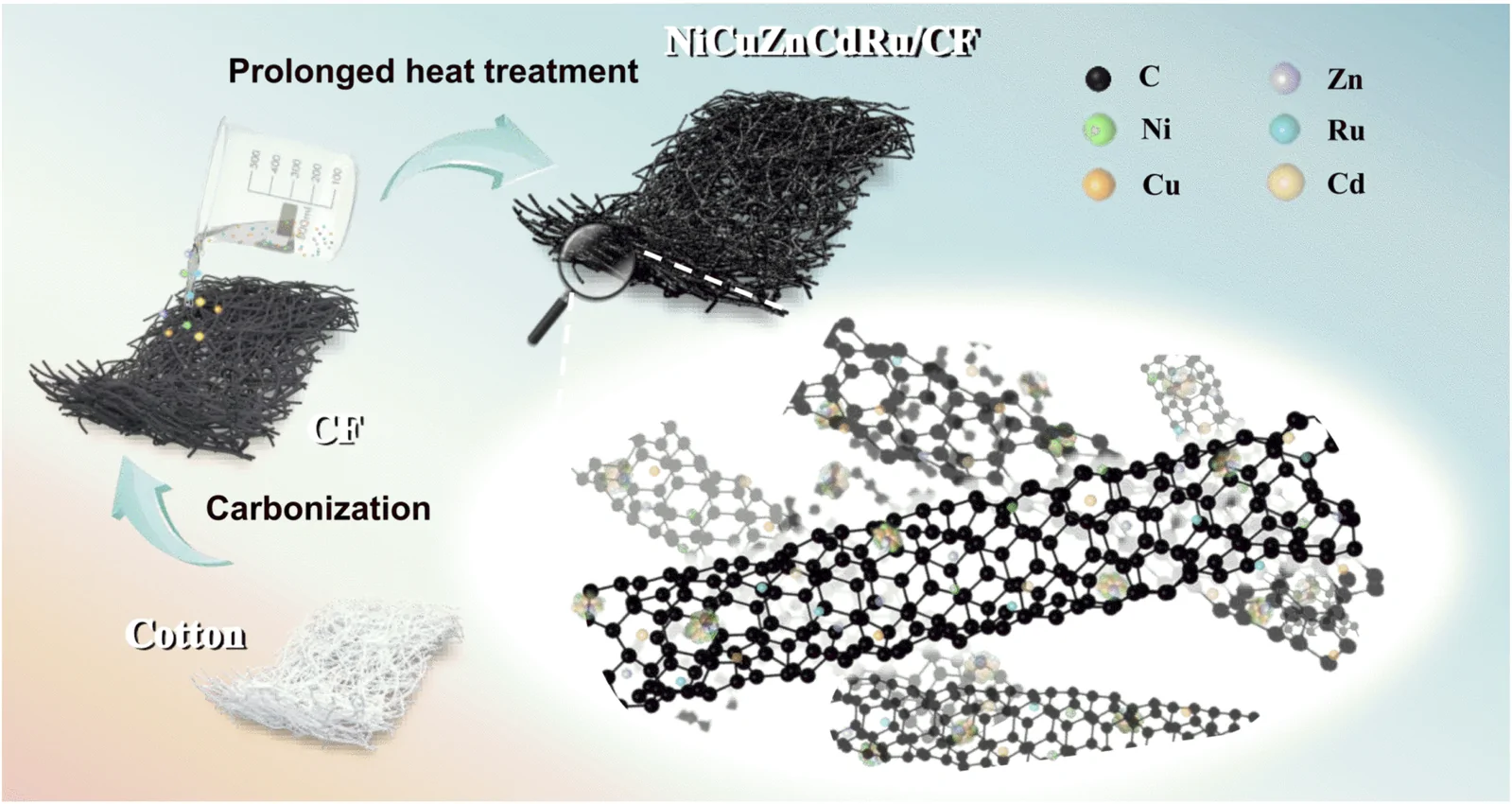
Synthesis scheme of the NiCuZnCdRu/CF cathode catalyst.

The CF substrate exhibits a layered morphology with distinct surface folds (Fig. S1). TEM images (Fig. 2a and S2) could observe the uniformly dispersed NiCuZnCdRu nanoparticles on the CF matrix. The pores in the layered structure can serve as transport channels, facilitating the diffusion of CO_2_ and electrolyte between the interior and exterior of the fibers.^24^ Meanwhile, the confinement effect of the CF matrix facilitates the uniform dispersion of metal nanoparticles and inhibits further migration and agglomeration. The atomic structure of the NiCuZnCdRu nanoparticles could be confirmed in the high-resolution image. A fast Fourier transform (FFT) analysis of this region confirms a face-centered cubic (FCC) like local ordering structure (Fig. 2b). The (111), (200), and (220) planes were identified and corresponding lattice spacings were measured to be 2.01 Å, 1.75 Å, and 1.34 Å, respectively. The FCC structure has the characteristics of stability, high density and adjustable expansion, ensuring the excellent structural integrity of NiCuZnCdRu/CF under electrochemical conditions, which may be responsible for its continued catalytic activity.^16^

**Fig. 2:**
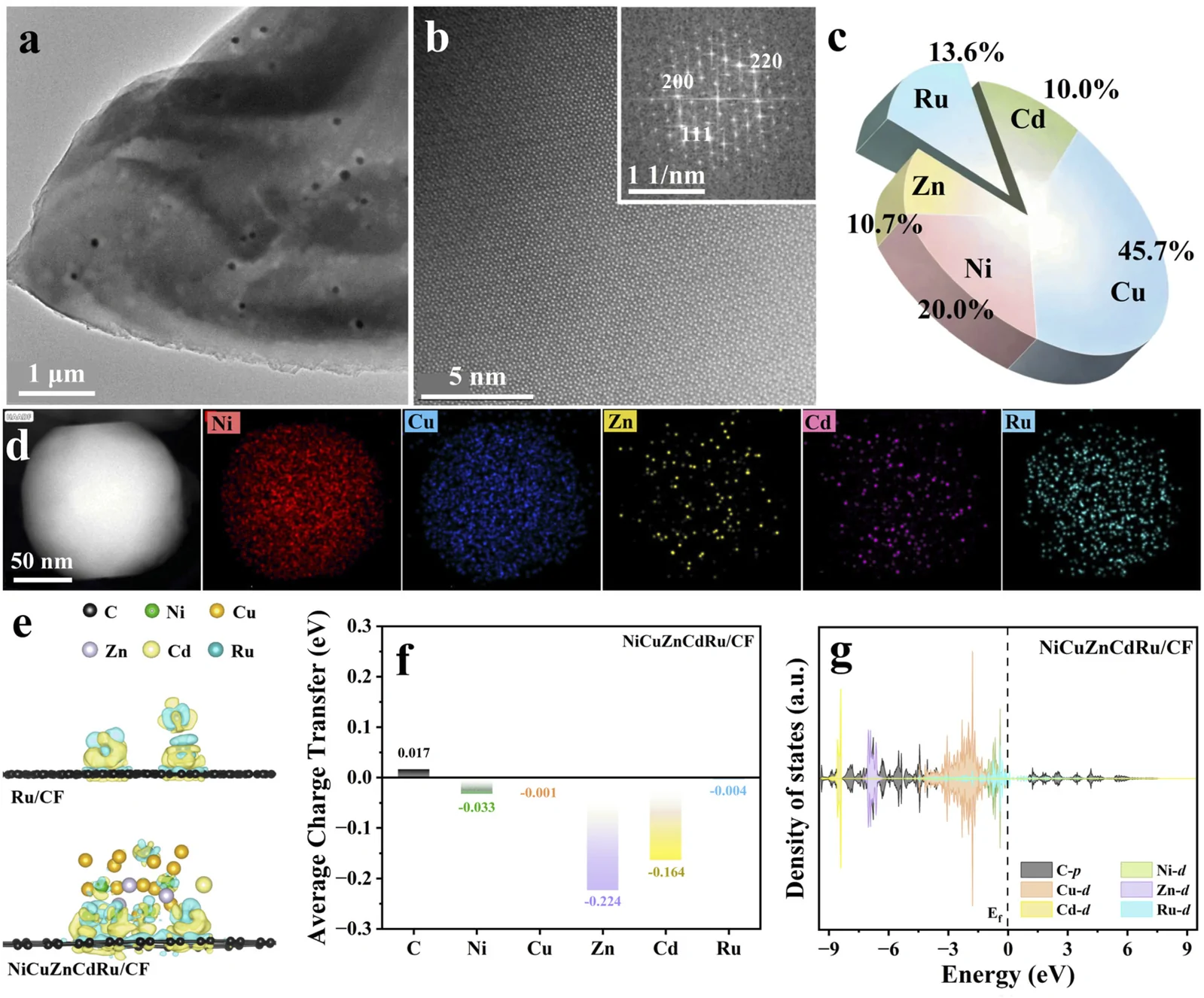
(a) and (b) TEM and HRTEM images of NiCuZnCdRu/CF. (c) Composition analysis by ICP-MS. (d) Corresponding element mapping of Ni, Cu, Zn, Cd, and Ru. (e) Charge density differences plots for Ru/CF and NiCuZnCdRu/CF; yellow and cyan regions represent charge accumulation and depletion, respectively. (f) Bader charge analysis of NiCuZnCdRu/CF. (g) Partial density of states curves of NiCuZnCdRu/CF.

The metal loading on carbon fibers was calculated to be 0.014 wt% based on the results from inductively coupled plasma mass spectroscopy (Table S1). Furthermore, the corresponding elemental ratio of Ni : Cu : Zn : Cd : Ru is 20.0 : 45.7 : 10.7 : 10.0 : 13.6 (Fig. 2c). The uniform elemental distribution is confirmed by the EDS mapping as shown in Fig. 2d. The XRD pattern shows distinctive peaks at 24.5° and 44.5° which could be ascribed to the (002) and (100) planes of biomass-derived carbon (Fig. S3).^25^ There were no evidence peaks from crystalline metallic phases owing to their low content. A similar phenomenon was reflected in the XPS results, where only C and O elements were identified (Fig. S4). The high-resolution C 1s spectrum exhibited three distinct peaks at 284.8, 286.0, and 289.0 eV, corresponding to the C–C, C–O, and C=O bonds, respectively. The peaks of the O 1s spectra at 532.5 and 533.7 eV are attributed to –OH and –COOH. Synchrotron X-ray absorption spectroscopy was employed to probe the local environment of metal active sites (Zn K-edge, Fig. S5 and Table S2). The spectrum of NiCuZnCdRu/CF differs from those of Zn foil and ZnO. EXAFS fitting identifies Zn–C and Zn–M scattering contributions with fitted distances of approximately 1.90 and 2.62 Å, respectively. According to the fitting results, Zn adopts a unique electronic environment resulting from its interaction with the neighboring metal atoms and the carbon support rather than a simply metallic phase.^7^

The Raman spectra exhibit characteristic D bands (1345 cm^−1^, disordered carbon) and G bands (1595 cm^−1^, graphitic carbon). The corresponding *I*_D_/*I*_G_ intensity ratios of NiCuZn/CF, NiCuZnCd/CF, and NiCuZnCdRu/CF are 1.29, 1.27, and 1.27, respectively, suggesting the defect density of the CF matrix is comparable (Fig. S6).^3^ The N_2_ adsorption–desorption curves present type I isotherms and H4 hysteresis loops, suggesting the coexistence of microporous and mesoporous structures (Fig. S7). NiCuZnCdRu/CF exhibits a high specific surface area of 1089.3 m^2^ g^−1^ and a pore volume of 0.42 cm^3^ g^−1^, which could enable reduced interfacial resistance and facilitated mass transfer during the CO_2_ conversion reactions.^24^

To elucidate the potential synergistic mechanisms among the multiple components, further density functional theory (DFT) calculations were performed. Fig. 2e presents the charge density distributions of Ru/CF and NiCuZnCdRu/CF. In contrast to the faint charge signal changes at the Ru–CF interface, NiCuZnCdRu/CF exhibits clear charge transfer between the CF matrix and the multi-metal nanoparticles. The distinct regions of charge accumulation and depletion surrounding the metal atoms confirm electron exchange and redistribution among these multiple metals. Bader charge analysis reveals the charge transfer mechanism within NiCuZnCdRu/CF (Fig. 2f). CFs act as electron donors, while Zn and Cd act as major electron acceptors which effectively attenuate the electron enrichment into Ru. As a result, the charge change of Ru is significantly reduced from −0.199 *e* to −0.004 *e*, realizing regulation of the electron density at the active sites. The density of states analysis in Fig. 2g and S8 also confirms the above electron interactions. PDOS analysis further reveals that the electronic states near the Fermi level are mainly contributed by the d-orbitals of Cu, Ni and Ru. Meanwhile, obvious overlap and hybridization of multi-metal d-orbitals could be observed, verifying the existence of multi-metal induced electronic redistribution. Element-resolved orbital analysis gives d-state centres of −1.93, −1.97, −5.06, −8.13, and −3.47 eV for Ni, Cu, Zn, Cd, and Ru, respectively. TDOS shows a high density of states at the Fermi level, confirming the excellent intrinsic metallic conductivity and its capability for multi-active-site cooperative catalysis in surface adsorption.^26^

### Electrochemical performance

The electrochemical performance of these cathodes for Li–CO_2_ batteries was assessed by cyclic voltammetry in a CO_2_ atmosphere within the voltage range of 2.0 to 4.5 V (Fig. 3a). NiCuZnCdRu/CF exhibits characteristic LCB redox peaks corresponding to CO_2_ reduction and Li_2_CO_3_ decomposition, and delivers a higher peak current density during the redox process than NiCuZnCd/CF and NiCuZn/CF.^1,27^ The maximum discharge/charge capacity tests at 20 µA cm^−2^ were employed to determine the maximum capacity of different cathodes (Fig. 3b). NiCuZnCdRu/CF delivers the highest specific discharge capacity (1949 µAh cm^−2^), outperforming the NiCuZnCd/CF (1226 µAh cm^−2^) and NiCuZn/CF (1169 µAh cm^−2^) cathodes. The outstanding capacity can be attributed to multi-metallic sites which could modulate electronic structures and intermediate adsorption, lower the reaction energy barriers, and mitigate the accumulation of insulating discharge products.^7^ A discharge voltage plateau as high as 2.75 V could be observed together with a largely reduced charge voltage, meaning that the CO_2_ redox kinetics are enhanced and superior reversibility is consequently achieved.

**Fig. 3:**
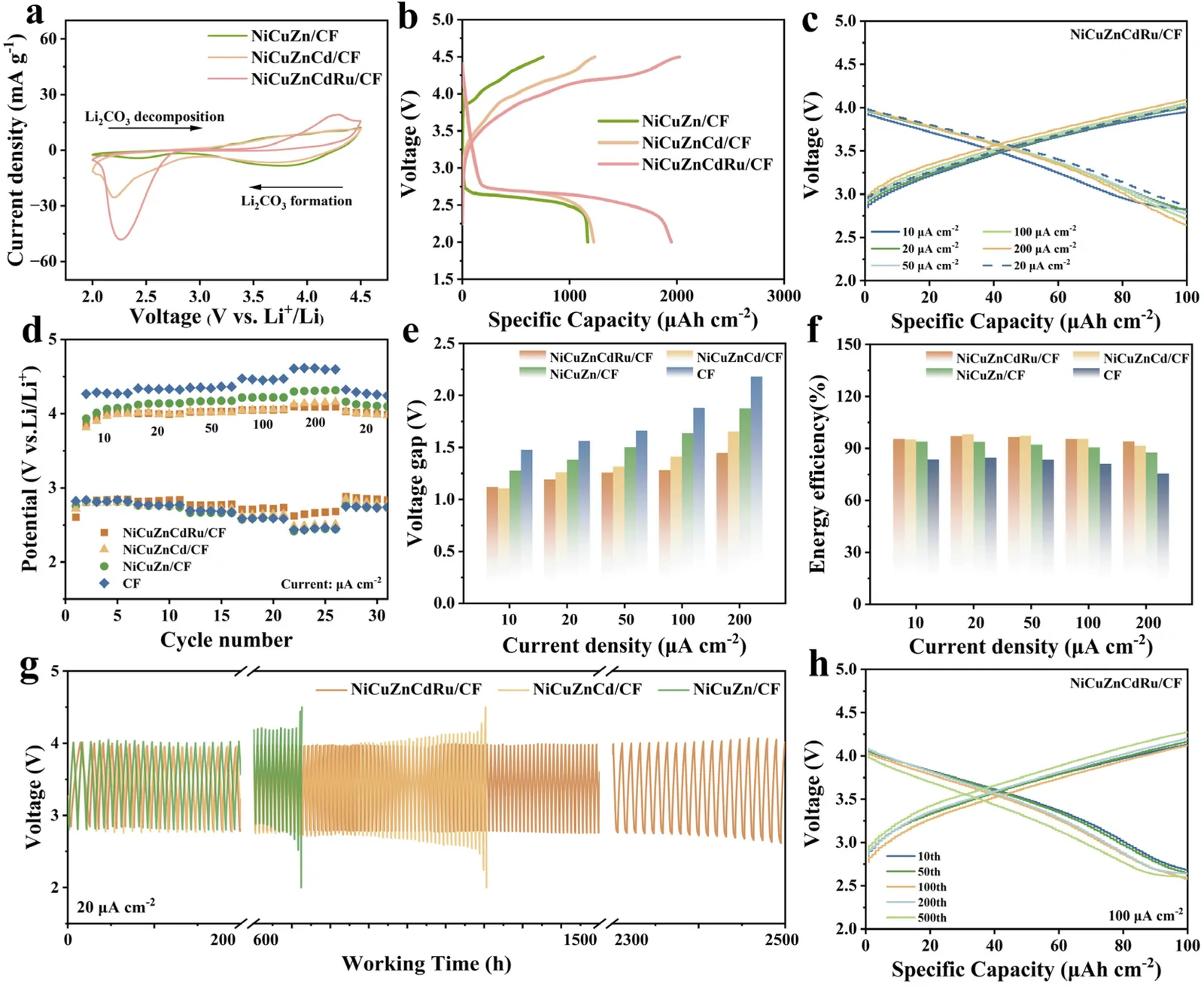
Evaluation of electrochemical performance. (a) CV curves at 0.1 mV s^−1^. (b) Maximum capacity profiles at 20 µA cm^−2^. (c) Galvanostatic discharge–charge profiles of NiCuZnCdRu/CF at different current densities. (d)–(f) End voltage, voltage gap and energy efficiency for the four cathodes from 10 to 200 µA cm^−2^. (g) Cycling stability of the NiCuZn/CF, NiCuZnCd/CF, and NiCuZnCdRu/CF cathodes at 20 µA cm^−2^. (h) Discharge–charge profiles of NiCuZnCdRu/CF at 100 µA cm^−2^.

As shown in Fig. 3c and S9, the NiCuZnCdRu/CF cathode exhibited well-defined discharge/charge plateaus and stable charge profiles over a range of current densities from 10 to 200 µA cm^−2^, suggesting rapid Li_2_CO_3_ redox kinetics even under high-rate conditions.^28^ The discharge voltages decrease from 2.82 V to 2.61 V as the current density increased, while the charge voltage increased from 3.95 V to 4.09 V. By contrast, both NiCuZn/CF and NiCuZnCd/CF cathodes show significantly higher polarization across all current densities (Fig. 3d). Notably, the NiCuZnCdRu/CF electrode exhibits a minimal voltage gap of 1.45 V at 200 µA cm^−2^, substantially narrower than that of pristine CF (2.18 V). Upon returning the current density to 20 µA cm^−2^, the discharge and charge voltages were restored to their original values, demonstrating excellent electrochemical reversibility. The details of electrochemical performance could be found in Fig. 3e, f and Table S3. The current densities range from 20 to 200 µA cm^−2^ while the smallest voltage gaps (1.12–1.45 V) and the highest energy efficiencies (93.9–97.2%) were delivered by the NiCuZnCdRu/CF cathode. Comparison between the literature studies in Table S4 confirms its outstanding performance, particularly in attaining a reduced voltage gap and improved energy efficiency achieved under identical test parameters.

Fig. 3g evaluates the cycling stability of these cathodes at 20 µA cm^−2^ and a curtailing capacity of 100 µAh cm^−2^. The NiCuZnCdRu/CF cathode demonstrates outstanding cycling stability over 250 cycles with a well-defined voltage plateau, which confirms remarkable structural reversibility of the catalyst. The NiCuZnCdRu/CF electrode also maintains the narrowest voltage gap of 1.26 V after 2500 h. Under identical testing conditions, the polarization levels of other cathodes are significantly higher, and their cycle times are notably shorter. Further tests at higher current densities (Fig. 3h, S10 and S11) showed that the NiCuZnCdRu/CF cathode could sustain 1100 h even at 100 µA cm^−2^, with an energy efficiency as high as 91.6%. The multi-metallic active sites regulate the local electronic structure and interfacial reaction kinetics, while the hierarchical CF framework facilitates mass transport and accommodates the discharge products.

By contrast, the electrochemical performance of CF-*T* (without multi-metallic components), Ru/CF (only the Ru element) and NiCuZnCdRu/CF-10 (higher loading content) is shown in Fig. S12. CF-*T* exhibits much larger charge–discharge polarization than that of NiCuZnCdRu/CF. Ru/CF presents a small initial voltage gap but the stability decays rapidly over 400 h. The NiCuZnCdRu/CF-10 electrode lasts about 800 h. Compared with the monometallic Ru catalyst, the charge redistribution and synergy effect within multiple elements enable rational modulation of the d band center and adsorption affinity toward reaction intermediates, overcoming the intrinsic limitation of single component Ru with insufficient tunability of intermediate binding strength. Benefiting from the structural robustness induced by the CF matrix and alloy effect, NiCuZnCdRu/CF could mitigate catalyst agglomeration, oxidation and dissolution upon cycling, achieving higher discharge capacity and longer cycling lifespan than bare Ru. Excessive loading catalysts lead to deteriorated electrocatalytic performance for Li CO_2_ batteries. High catalyst loading induces agglomeration of nanoparticles, and a decrease in the accessible specific surface area. Meanwhile, over loading blocks the pore channels of the cathode host, increasing the mass transport resistance for CO_2_, electrolyte and Li^+^. Moreover, excessive stacking of catalyst raises the charge transfer impedance, resulting in increased overpotential, reduced discharge capacity and degraded cycling stability.

### Reversibility and stability of Li–CO_2_ batteries

A reversible conversion of discharge products is critical for maintaining the stable operation of Li–CO_2_ batteries.^26^ To clarify the reaction pathway of the NiCuZnCdRu/CF cathode in Li–CO_2_ batteries, *ex situ* characterization studies were conducted at different cycling stages and constant current density to track its morphological and composition evolution (Fig. 4 and S13). As shown in Fig. 4a and S14, at a discharge capacity of 400 µAh cm^−2^, the CF electrode already exhibits stacked deposition of uneven spherical particles. In contrast, the NiCuZnCdRu/CF electrode displays a uniform sheet-like structure of discharge products. This fine and uniform morphology not only helps alleviate volume expansion during cycling but also facilitates decomposition of the discharge products upon subsequent charging. After charging, the products on the NiCuZnCdRu/CF electrode surface almost completely disappear, and the electrode structure reverts to its original state, fully confirming the excellent reversibility and catalytic activity of this catalyst. The NiCuZnCdRu/CF cathode primarily directs the growth of discharge products through a surface adsorption mechanism. The multi-metal components modulate the electronic structure of the active sites, which promotes rapid electron transfer and substantially reduces residual product clogging in the electrode pores, thereby enhancing the overall electrochemical performance.^29^

**Fig. 4:**
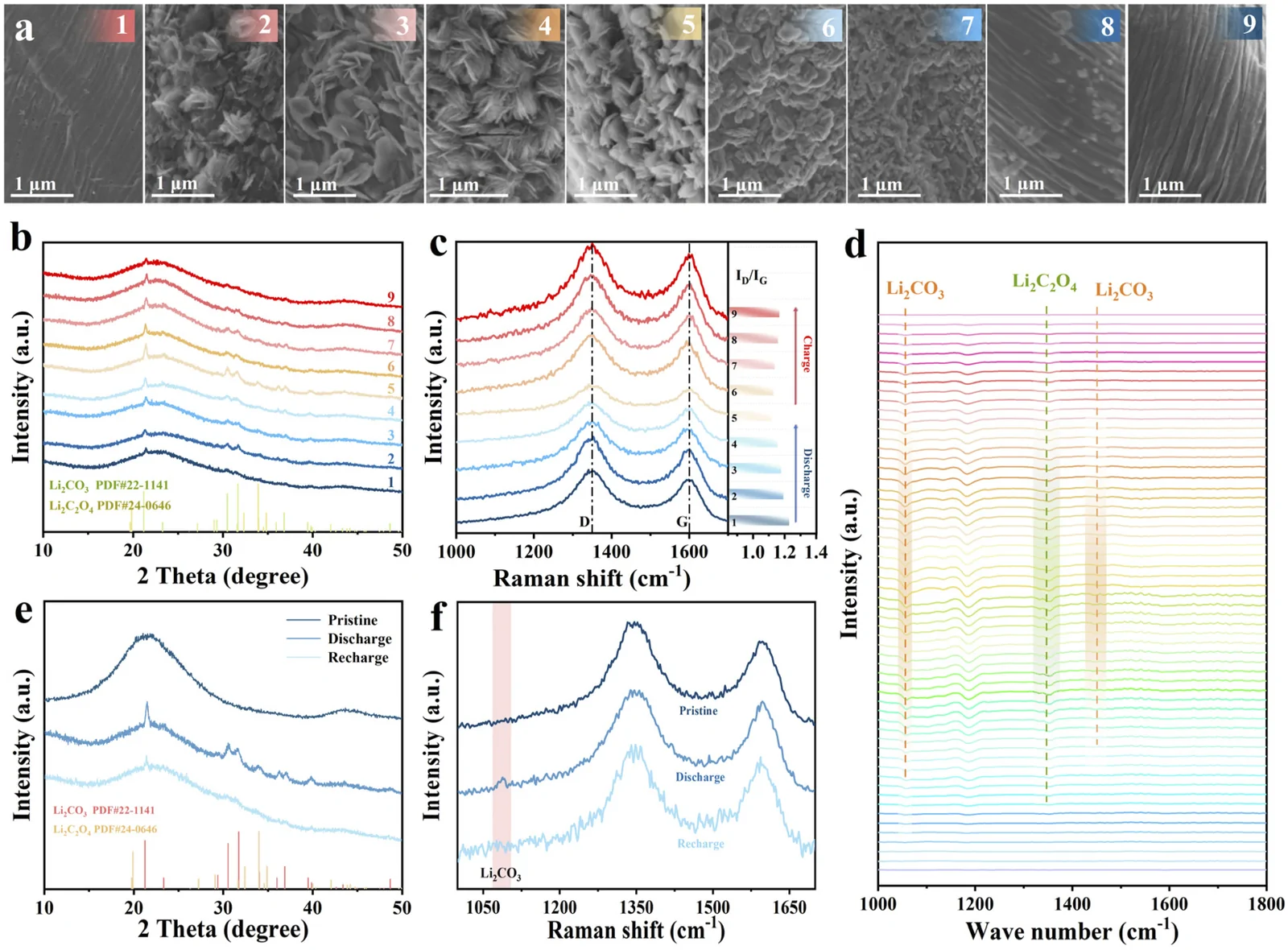
Characterization of the NiCuZnCdRu/CF cathode at various stages during discharge–charge in a CO_2_ atmosphere. *Ex situ* (a) SEM images, (b) XRD patterns, and (c) Raman spectra at a fixed discharge capacity of 800 µAh cm^−2^. (d) *In situ* FTIR spectra. (e) XRD patterns, and (f) Raman spectra of the Li–CO_2_ battery with the NiCuZnCdRu/CF cathode under the fully discharged and recharged states.

The substance transformation during cycling was observed by *ex situ* XRD analysis. Fig. 4b shows that characteristic peaks of Li_2_CO_3_ (PDF#22-1141) and Li_2_C_2_O_4_ (PDF#24-0646) appear upon the discharge process, and disappear at the charge terminal by virtue of the highly active NiCuZnCdRu/CF cathode.^30^ The *I*_D_/*I*_G_ ratio in Raman spectroscopy complementally reveals the reversible dynamic transformation of CF substances (Fig. 4c).^2,20^ These findings demonstrate the effectiveness of the NiCuZnCdRu/CF cathode in optimizing the evolution behavior of Li_2_CO_3_ and C, thereby lowering the charge overpotential and increasing the energy efficiency of the battery. This is further supported by *in situ* FT-IR spectroscopy (Fig. 4d). The FT-IR signals were normalized to minimize interference from other species on the electrode surface. The peak at 1350 cm^−1^ is assigned to Li_2_C_2_O_4_, while the bands at 1055 and 1450 cm^−1^ correspond to Li_2_CO_3_.^14,31^ During discharge, the intensities of these characteristic peaks gradually increase, indicating the formation and accumulation of both products. Upon charging, the signals progressively weaken, confirming the reversible nature of the reaction.

The morphological evolution of the electrode surface during deep charge–discharge was further systematically studied. The XRD patterns (Fig. 4e) show diffraction peaks assigned to Li_2_CO_3_ and Li_2_C_2_O_4_ in the discharged states, while Raman spectra (Fig. 4f) clearly depicted a characteristic Li_2_CO_3_ peak at about 1085 cm^−1^.^32,33^ The near-total disappearance of this spectral signal after charging corresponds to the effective promotion of reversible crystallization and subsequent decomposition of the discharge products by the NiCuZnCdRu/CF catalyst. Reversible morphological evolution at the cathode side was clearly shown in SEM images across the whole cycle (Fig. S15). A densely distributed and fluffy product overlay is formed on the surface of the electrode after deep discharge. Following the subsequent charge process, this layer is almost entirely removed, indicating that the catalytic structure remains intact. High-resolution XPS analyses of the cathode in the pristine, discharged, and charged states further demonstrate the ability of the NiCuZnCdRu/CF catalyst to regulate the reaction pathway (Fig. S16). The characteristic peak centered at approximately 290.0 eV is attributed to carbonate species (CO_3_^2−^), indicative of Li_2_CO_3_ formation. Two distinct peaks are observed at binding energies of 284.8 eV and 286.8 eV, which are characteristic of C–C and C–O bonds, respectively. The signal near 293.0 eV originates from the –CF_3_ group in the electrolyte.^34^ The significant attenuation and shift of the C–O and Li_2_CO_3_-related signals after cycling clearly indicate the reversible electrochemical process.^22,35^

Variations in electrochemical behavior among batteries are primarily attributed to evolving interfacial mass-transfer kinetics, monitored *via in situ* electrochemical impedance spectroscopy (EIS). Mass transfer is adversely affected by the nonuniform deposition of discharge products.^36^ The typical Nyquist plots show a semicircle in the high and mid-frequency regions, corresponding to charge transfer processes, and a linear segment in the low-frequency region, associated with ion diffusion.^37^ Fig. S17 tracks the impedance evolution of CF, NiCuZnCd/CF, and NiCuZnCdRu/CF cathodes at the same voltage limit, illustrating the formation and decomposition of discharge products upon cycling and their impact on electrochemical behavior. The NiCuZnCdRu/CF cathode exhibits the smallest impedance change, highlighting its outstanding electrochemical stability.

Electrochemical impedance data were analyzed using the DRT method to gain mechanistic insight into the kinetic process (Fig. 5a–d and S18). A characteristic time constant (*τ*) is assigned to each identified polarization process, serving as a quantitative measure of its contribution to the overall polarization resistance.^37^ The process in the time constant range of 10^−6^ to 10^−4^ s corresponds to the transport of Li^+^ in the liquid electrolyte and initial charge transfer at the electrode electrolyte interface, which is represented as a high-frequency semicircle in the Nyquist plot. The characteristic time distribution of the charge transfer process associated with redox reactions ranges from 10^−4^ to 10^−2^ s, ascribed to the electrochemical reactions identified in the mid-frequency semicircle region.^38,39^ Peaks with time constants of 10^−1^ to 10^2^ s correspond to the interfacial diffusion process, forming the low-frequency region of the Nyquist plot.

**Fig. 5:**
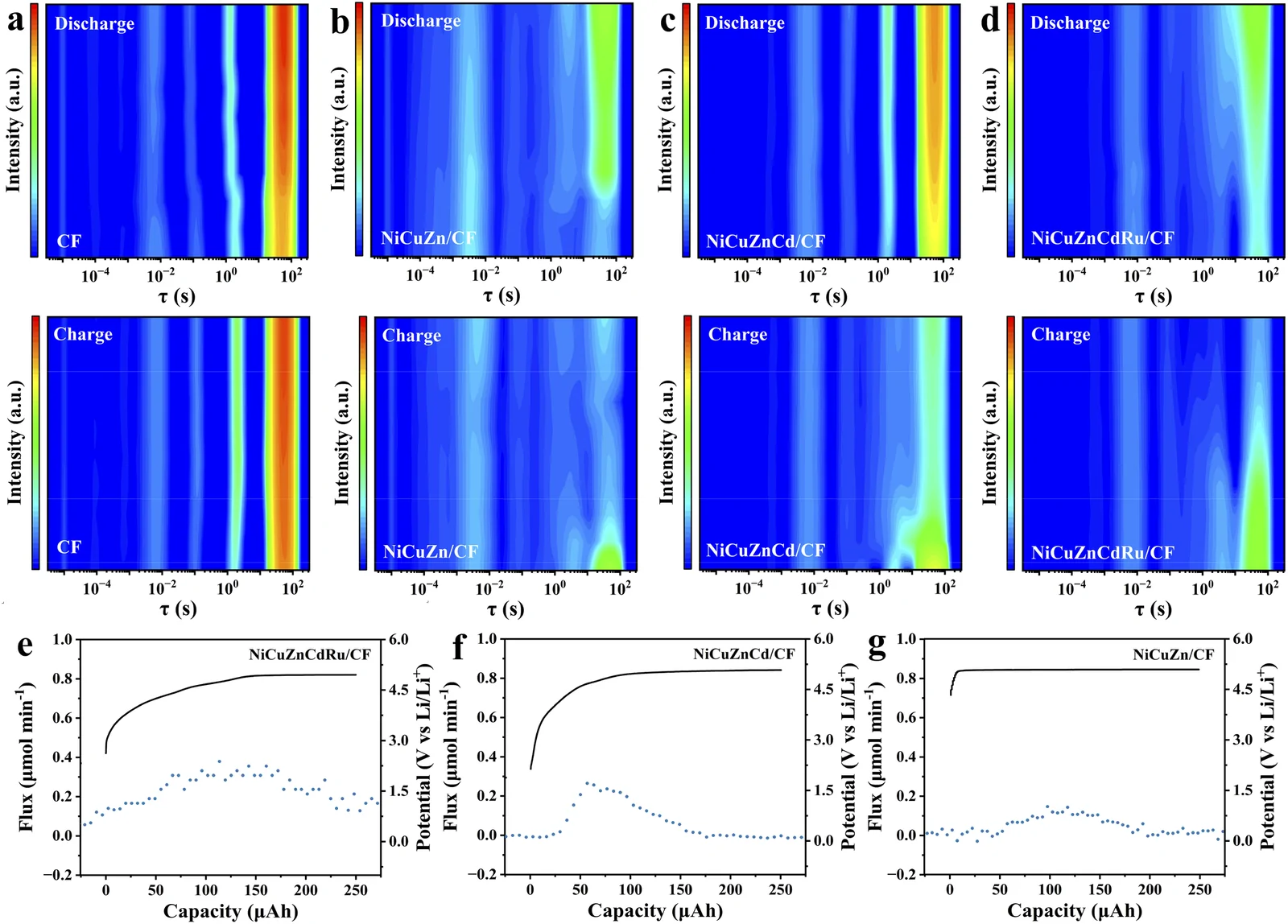
(a)–(d) 2D intensity color maps of the DRT derived from EIS measurements for the cathodes at different discharge–charge voltages. (e)–(g) DEMS results of the NiCuZnCdRu/CF, NiCuZnCd/CF, and NiCuZn/CF electrodes in charge processes at 500 µA.

Compared with the pristine CF electrode, the introduction of catalysis active sites could effectively reduce the interfacial diffusion resistance. The monometallic Ru/CF electrode only exhibits superiority in lowering the impedance during the charging process. A comparison of the NiCuZn/CF and NiCuZnCd/CF electrodes reveals that the incorporation of Cd lowers the charge-transfer resistance, albeit with a slight hindrance to Li^+^ diffusion. Upon further Ru incorporation, both the charge-transfer resistance and low-frequency impedance response are concurrently suppressed. In addition, the impedance evolution over discharge–charge cycling exhibits improved symmetry. The improved reversibility of discharge–charge evolution indicates suppressed interfacial passivation, reduced product-layer transport resistance, and more effective restoration of the cathode during the charging process, which is consistent with the excellent long-term cycling stability of NiCuZnCdRu/CF. Collectively, these results demonstrate that the synergistic interplay among multiple metal components optimizes and indirectly mitigates low-frequency transport limitations by regulating the nucleation, spatial distribution, and decomposition of the solid products.^40,41^

*In situ* DEMS was employed to monitor CO_2_ evolution of NiCuZnCdRu/CF, NiCuZnCd/CF, and NiCuZn/CF cathodes. As shown in Fig. 5e–g, the moles of CO_2_ released were quantified by integrating the DEMS signal over time (Table S5). During the charging process, the CO_2_ evolution on the NiCuZnCdRu/CF electrode was much higher than that of the NiCuZnCd/CF and NiCuZn/CF electrodes. The measured CO_2_ amounts were 5.9, 5.4, and 3.2 µmol for the three cathodes, with corresponding conversion efficiencies of 84.3%, 77.7%, and 45.4%, respectively.^42^

Density-functional-theory (DFT) calculations were further employed to investigate the modulation effect of multiple metal components on intermediate species and reaction pathways in Li–CO_2_ batteries (Fig. 6b and S19). NiCuZnCdRu/CF displays higher adsorption energies than CFs for Li, CO_2_, and Li_2_CO_3_, indicating superior affinity that promotes mass transfer. In addition, the optimized electronic structure and active catalytic interface facilitate efficient Li_2_CO_3_ decomposition. Subsequently, the possible reaction pathways of Li–CO_2_ batteries on CF, Ru/CF, and NiCuZnCdRu/CF electrodes were calculated to gain a deeper understanding of the origins of their performance differences (Fig. 6c and S20). The calculated results reveal that the rate-determining step for all three electrodes involves the formation of C, with energy barriers of 2.80, 2.31, and 1.83 eV, respectively. Among them, NiCuZnCdRu/CF exhibits the lowest energy barrier, which facilitates the nucleation and growth of Li_2_CO_3_ and C. Importantly, the synergistic effect of the multiple metallic components facilitates the formation of key intermediates with Li^+^, which consequently reduces the reaction barrier. The difference in charge density of key intermediate compounds (Fig. S21) shows electron accumulation primarily around the adsorbed C/O-containing intermediates and electron depletion over multiple metal and carbon sites. The spatially distributed response indicates that adsorption is not localized exclusively on an isolated Ru atom. Bader analysis further reveals the metal specific and intermediate dependent charge rearrangement (Table S6).

**Fig. 6:**
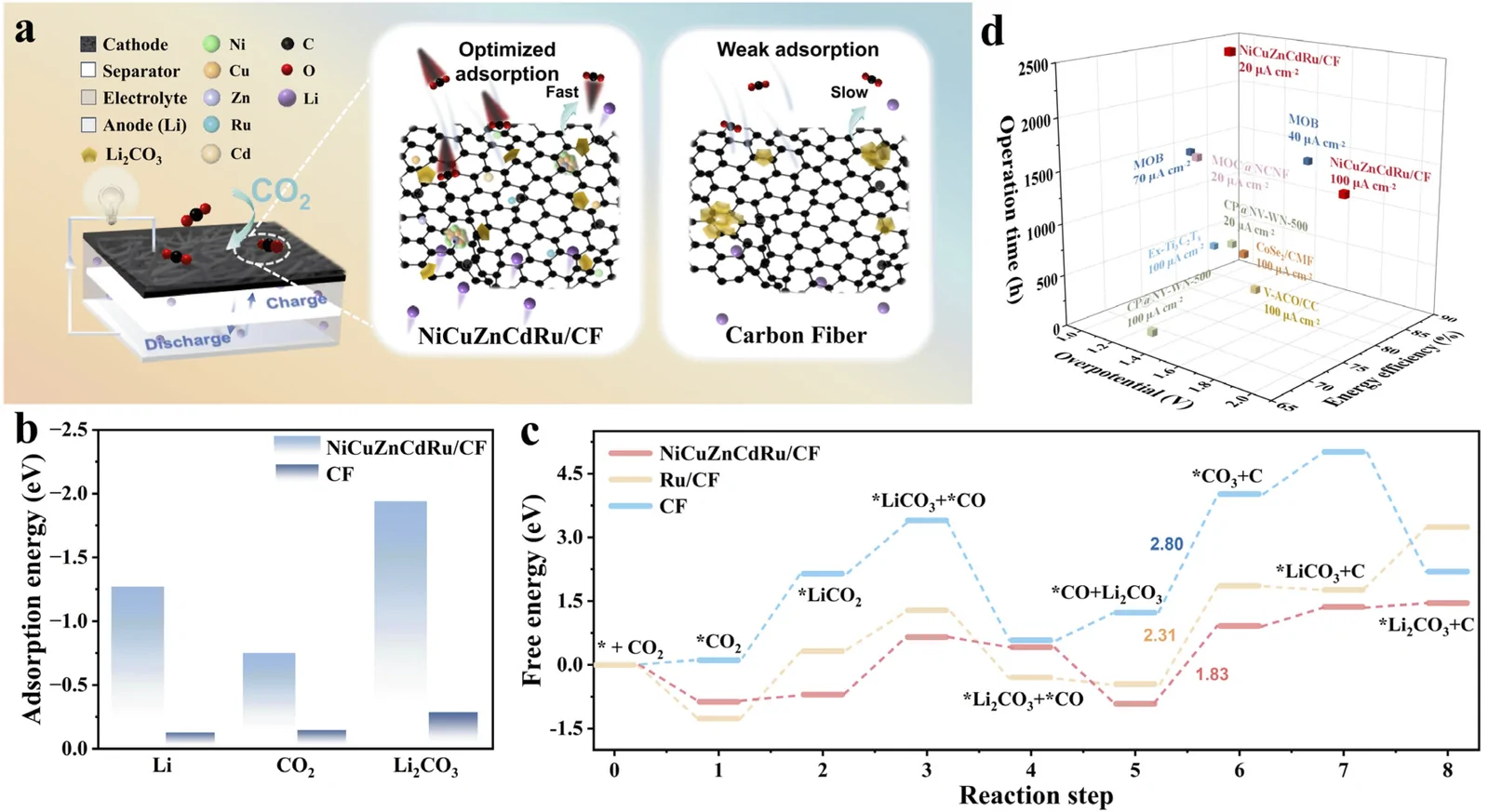
(a) Schematic diagram illustrating the configuration of a Li–CO_2_ battery utilizing NiCuZnCdRu/CF as the cathode catalyst. (b) Calculated adsorption energies of Li, CO_2_, and Li_2_CO_3_ on CF and NiCuZnCdRu/CF. (c) Comparison of the free energy diagram of CO_2_ to Li_2_CO_3_ on CF, Ru/CF, and NiCuZnCdRu/CF. (d) Comparative performance analysis with other reported catalysts.

Fig. 6a schematically presents the catalytic mechanism of the NiCuZnCdRu/CF cathode involving an accelerated mass transfer process for homogenization of discharge product growth. The charge redistribution among multi-metallic NiCuZnCdRu nanoparticles modulates the d-band center and precisely tunes the adsorption strength of key intermediates, achieving high catalytic activity, fast reaction kinetics at high current density, high energy efficiency and excellent cycle stability.

To further evaluate the overall electrochemical competence of the NiCuZnCdRu/CF cathode for Li–CO_2_ batteries, its key performance metrics including discharge–charge overpotential, energy efficiency and long-term cyclability have been compared with those of previously reported cathode catalysts (Fig. 6d, S22 and Table S7). The exceptional long-term cycle performance surpasses that of previously reported systems. Additionally, the NiCuZnCdRu/CF cathode shows sparse discharge-product particles and retains structural integrity after 2000 h operation, indicating suppressed product accumulation and outstanding electrochemical reversibility.^43–48^

## Conclusions

In summary, the NiCuZnCdRu/CF cathode catalyst was synthesized *via* a slow thermodynamically driven phase transition method. The multi-metal induced electronic regulation, accessible metal–carbon interfaces, particle dispersion, and confinement by the porous carbon framework generate abundant active sites for the complex CO_2_RR/CO_2_ER, which accelerates the transformation process and secures reversible Li_2_CO_3_ conversion. As a result, the NiCuZnCdRu/CF cathode achieves a low voltage gap of 1.19 V and a high energy efficiency of 97.2% along with exceptional durability over 2500 hours at 20 µA cm^−2^. After more than 500 stable cycles at 100 µA cm^−2^, the energy efficiency still remains above 91.6%. This study offers a feasible strategy for preparing durable cathodes in advanced Li–CO_2_ batteries with trace-level multi-metallic synergy.

## Author contributions

J. Sun conceived the idea and wrote the manuscript. Q. Jiang supervised the work and revised the manuscript. X. Li and G. Zeng analysed part of the data and contributed to the preparation of the manuscript. X. Han, Y. Liu, and T. Zhou contributed to the discussion and provided suggestions. J. Hu supervised the work and directed the project. All the authors discussed and analysed the results, and commented on the manuscript.

## Conflicts of interest

There are no conflicts to declare.

## Supplementary Material

SC-017-D6SC05155C-s001

## Data Availability

All data needed to evaluate the conclusions are present in the article and/or the supplementary information (SI). Supplementary information (SI) is available. See DOI: https://doi.org/10.1039/d6sc05155c.
