# Supplementary material for: Trace-level ruthenium incorporated with transition metal components enabled the development of high-efficiency Li–CO2 batteries
Source: Chem Sci. 2026 Aug 14;17(39):18817–26. doi: 10.1039/d6sc05155c (PMC13491321; doi:10.1039/d6sc05155c)
Supplement: Supplementary file 1 [file SC-017-D6SC05155C-s001.pdf]

Supplementary Information (SI) for Chemical Science.
This journal is © The Royal Society of Chemistry 2026

Supporting information for:

Trace-level Ruthenium Incorporated with Transition Metal Components Enabled High-Efficiency Li-CO_2 Battery

1. Supplementary Methods

1.1 Chemicals and Material

The cotton was purchased from Unicharm Lifestyle Products Co., Ltd. (China). Zinc nitrate ($Zn(NO_3)_2 \cdot 6H_2O$, ≥99.99%) and Ruthenium(III) chloride anhydrous (45-55% Ru content) were acquired from Shanghai Aladdin Biochemical Technology Co., Ltd. Nickel nitrate ($Ni(NO_3)_2 \cdot 6H_2O$, ≥98.0%), Copper nitrate ($Cu(NO_3)_2 \cdot 3H_2O$, 99.0~102.0%), Cadmium nitrate ($Cd(NO_3)_2 \cdot 4H_2O$, ≥99.0%) and Ethanol anhydrous (C_2H_6O) were provided by Sinopharm Chemical Reagent Co. Ltd. Carbon dioxide (CO_2, 99.999%) was ordered from Haohua Gas Co., Ltd. Wuhan Branch. Whatman separator (Grade GF/D) was bought from Global Life Sciences Solutions Operations UK Ltd. Electrolyte (LK-001) was purchased from Suzhou Duoduo Chemical Technology Co., Ltd. Lithium flakes were acquired from Xinfeng Lithium Co., Ltd. All the chemicals and solvents were used as received without further purification.

1.2 Materials Characterization

The morphology and microstructure of the synthesized materials were characterized by scanning electron microscopy (SEM, HITACHI SU-8010) and high-resolution transmission electron microscopy (HRTEM/STEM, JEOL JEM-2010 LaB6). The crystal structure was analyzed by X-ray diffraction (XRD, Rigaku SmartLab SE) at a scanning speed of 2° min^{-1}. Surface composition and elemental valence states were determined via X-ray photoelectron spectroscopy (XPS, Mutilab 2000) spectrometer. Raman spectra were acquired using a DXR confocal microscope (Thermo Fisher Scientific) with a 532 nm laser. The specific surface area was evaluated by Brunauer-Emmett-Teller (BET, Micromeritics ASAP 2460), and surface functional groups were identified by Fourier-transform infrared (FTIR, ALPHA) spectroscopy. Post-cycling characterization of cathode materials from disassembled batteries was performed using SEM, XRD, Raman spectroscopy, FTIR, and XPS.

1.3 DFT calculations

Theoretical calculations were performed using the Vienna Ab initio Simulation Package 6.4.3 (VASP 6.4.3) based on Density Functional Theory (DFT).[1–4] The exchange-correlation interactions were described by the Perdew-Burke-Ernzerhof (PBE) functional within the Generalized Gradient Approximation (GGA), while the DFT-D3 method was employed to account for long-range van der Waals interactions between the metal clusters and the graphene substrate.[5,6] The ion-electron interactions were treated using the projector augmented-wave (PAW) method with a plane-wave cutoff energy of 500 eV. For the structural optimization, the Brillouin zone was sampled with a Monkhorst-Pack k-point grid of 3x3x1, and the convergence criteria for energy and force were set to 10^{-5} eV and 0.02 eV/Å, respectively. Spin-polarization was considered throughout the calculations.

The adsorption energy (E_{ad}) of molecules was evaluated as:

$$E_{ad} = E_{sur+adsorbate} - E_{sur} - E_{adsorbate}$$

where $E_{sur+adsorbate}$ denotes the total energy of the adsorbed system, E_{sur} is the energy of surface, and $E_{adsorbate}$ represents the energy of adsorbate.

Post-processing of the data, including the extraction of the reaction Gibbs free energy and electronic properties, was performed using the VASPKIT (version 1.5.1) package. The charge density difference (Δρ) was calculated to visualize the electronic interactions between the metal clusters and the graphene carbon rings, defined as:

$$\Delta\rho = \rho_{total} - \rho_{cluster} - \rho_{graphene},$$

where ρ_{total}, $\rho_{cluster}$, and $\rho_{graphene}$ are the charge densities of the composite system, the isolated metal cluster, and the pristine graphene sheet in the same coordinate system, respectively.

The DFT model represents a simplified structural surrogate for the experimental NiCuZnCdRu/CF material and does not attempt to reproduce the exact morphology, coordination geometry, or absolute density of metal sites at the experimental loading. The relative numbers of Ni, Cu, Zn, Cd, and Ru atoms in the computational model were directly derived from the ICP-OES measured elemental ratio. The calculated charge-density differences, Bader charges, PDOS, and free-energy profiles should be interpreted as qualitative mechanistic trends rather than an exact atomistic reconstruction of the ultralow-loading experimental material.

2. Supplemental Figures

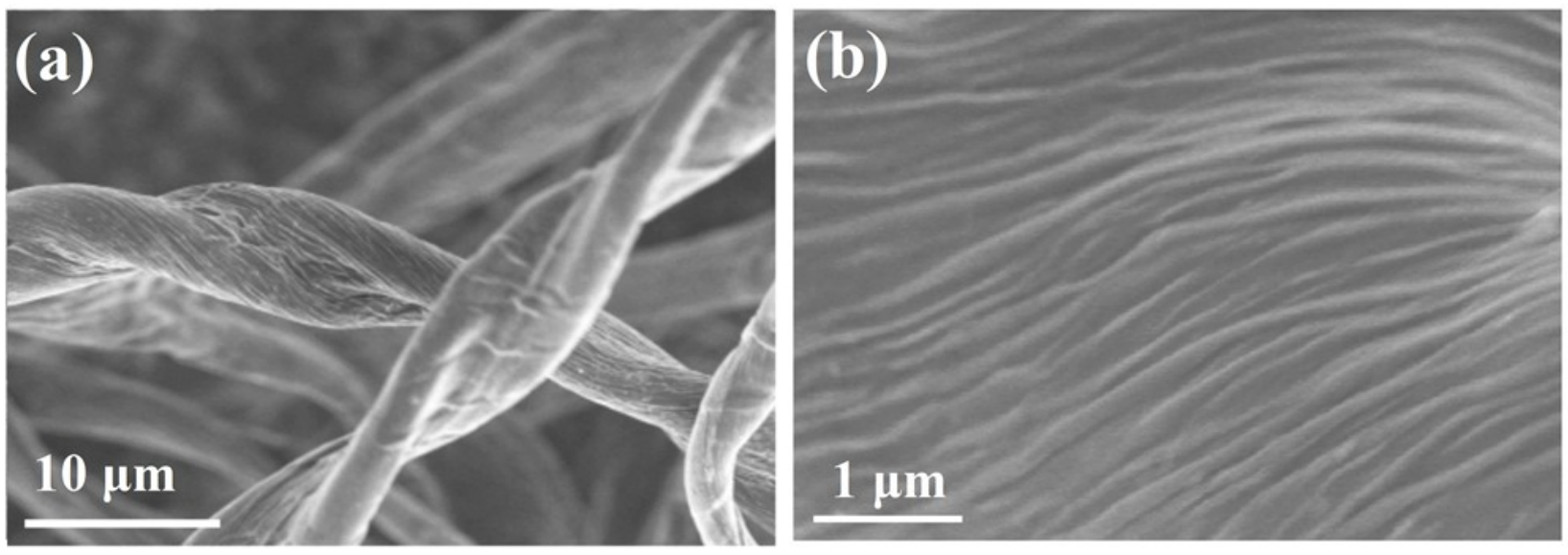

Fig. S1. SEM images of NiCuZnCdRu/CF.

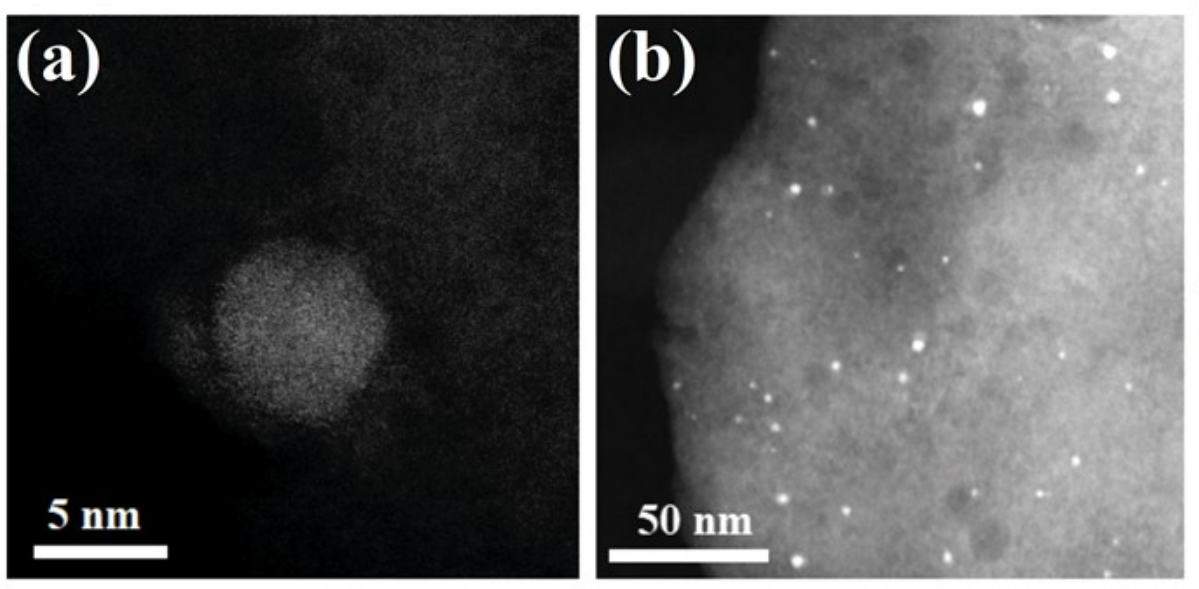

Fig. S2. HAADF-STEM image of the NiCuZnCdRu/CF.

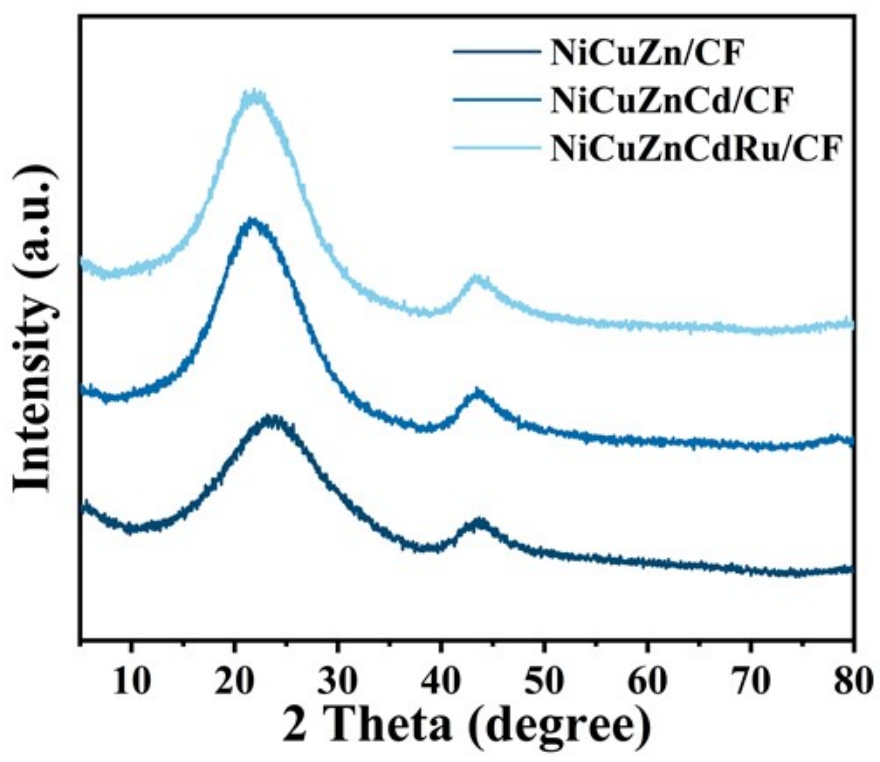

Fig. S3. XRD spectra of NiCuZn/CF, NiCuZnCd/CF, and NiCuZnCdRu/CF.

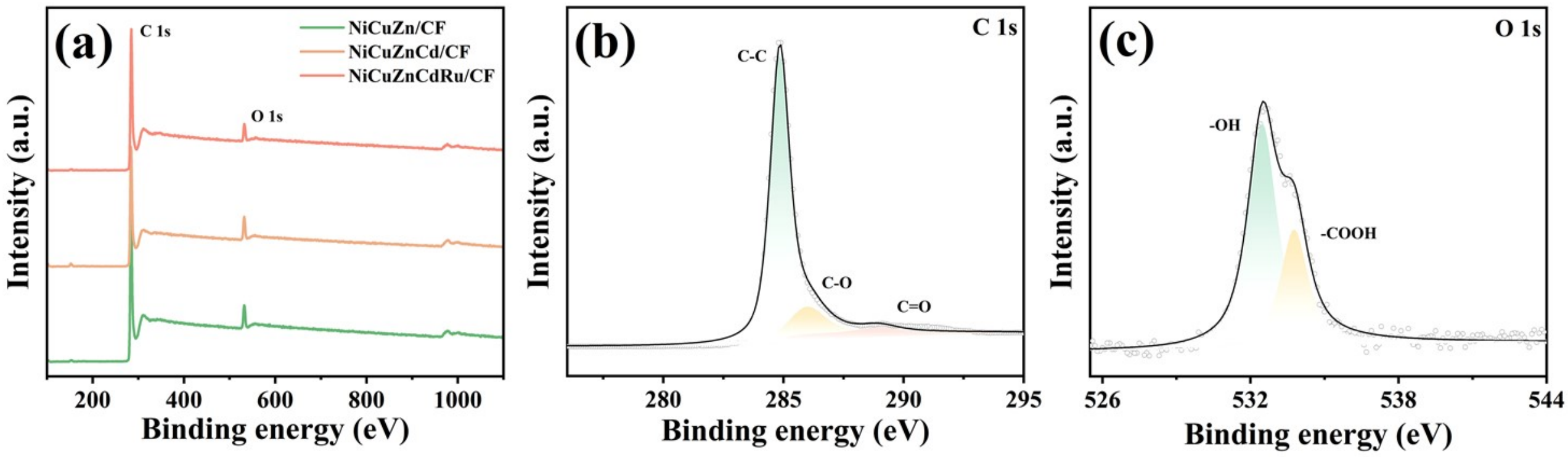

Fig. S4. (a) XPS survey results of NiCuZn/CF, NiCuZnCd/CF and NiCuZnCdRu/CF. High resolution XPS spectra of (b) C 1s, and (c) O 1s of the NiCuZnCdRu/CF.

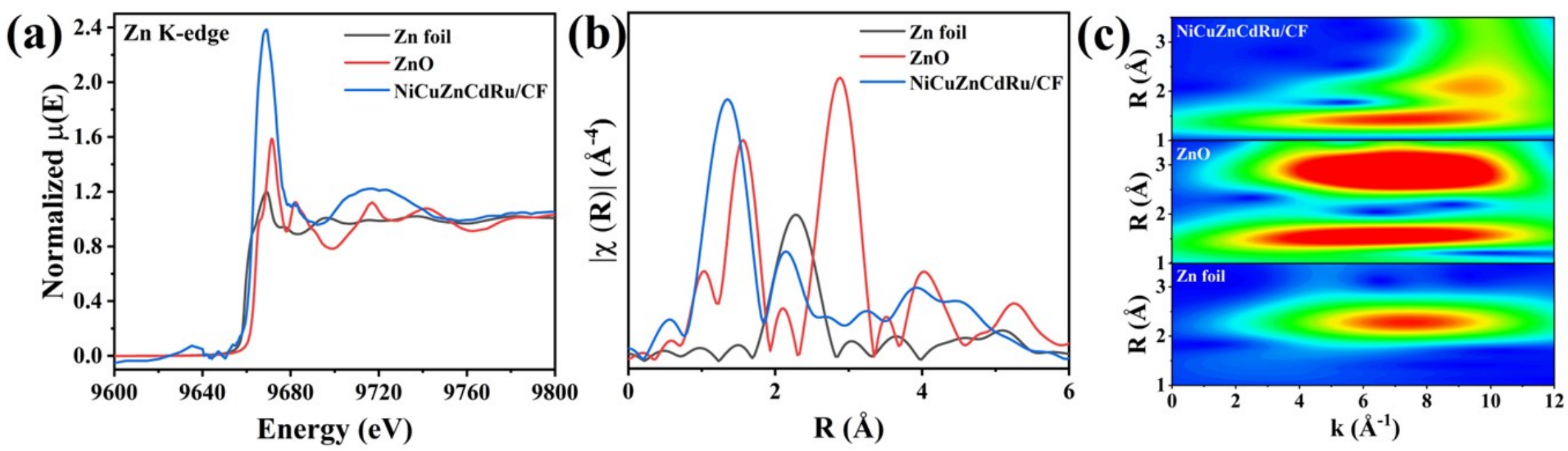

Fig. S5. (a) Zn K-edge XANES spectra, (b) Fourier transform (FT) of Zn K-edge extended X-ray absorption fine structure (EXAFS) spectra, and (c) Wavelet transformed (WT) of the FT-EXAFS of Zn in NiCuZnCdRu/CF.

Fig. S6. Raman spectra of (a) NiCuZn/CF, NiCuZnCd/CF, NiCuZnCdRu/CF, and (b) CF.

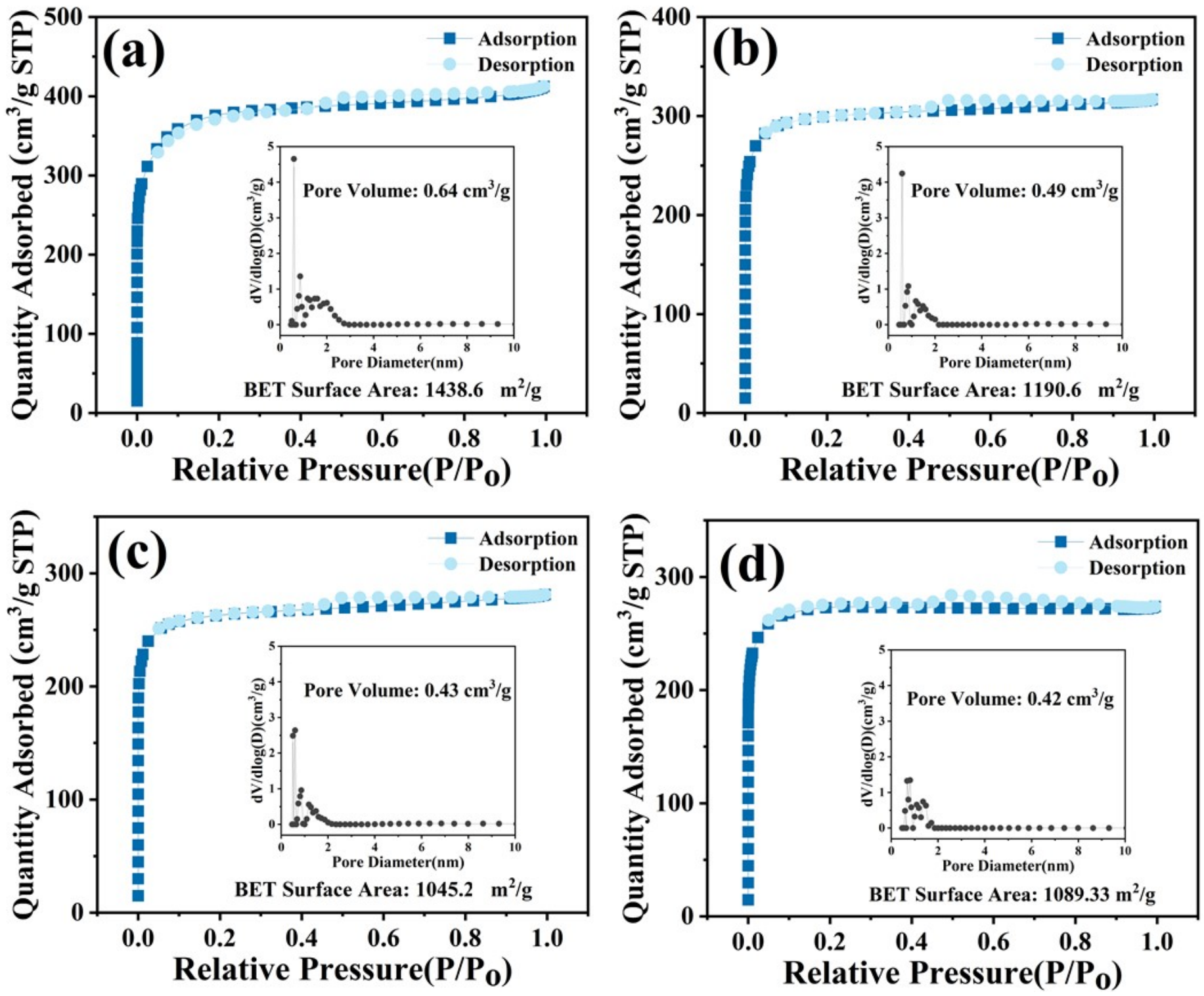

Fig. S7. Nitrogen sorption isotherms and pore size distribution profiles (inset) of (a) CF, (b) NiCuZn/CF, (c) NiCuZnCd/CF, and (d) NiCuZnCdRu/CF.

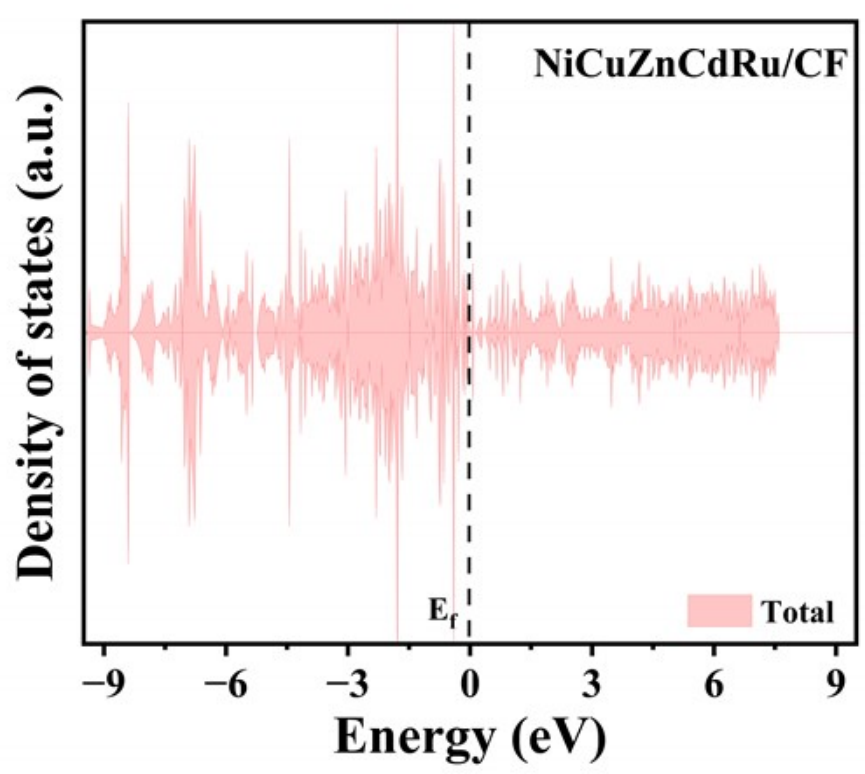

Fig. S8. Total density of states curves of NiCuZnCdRu/CF.

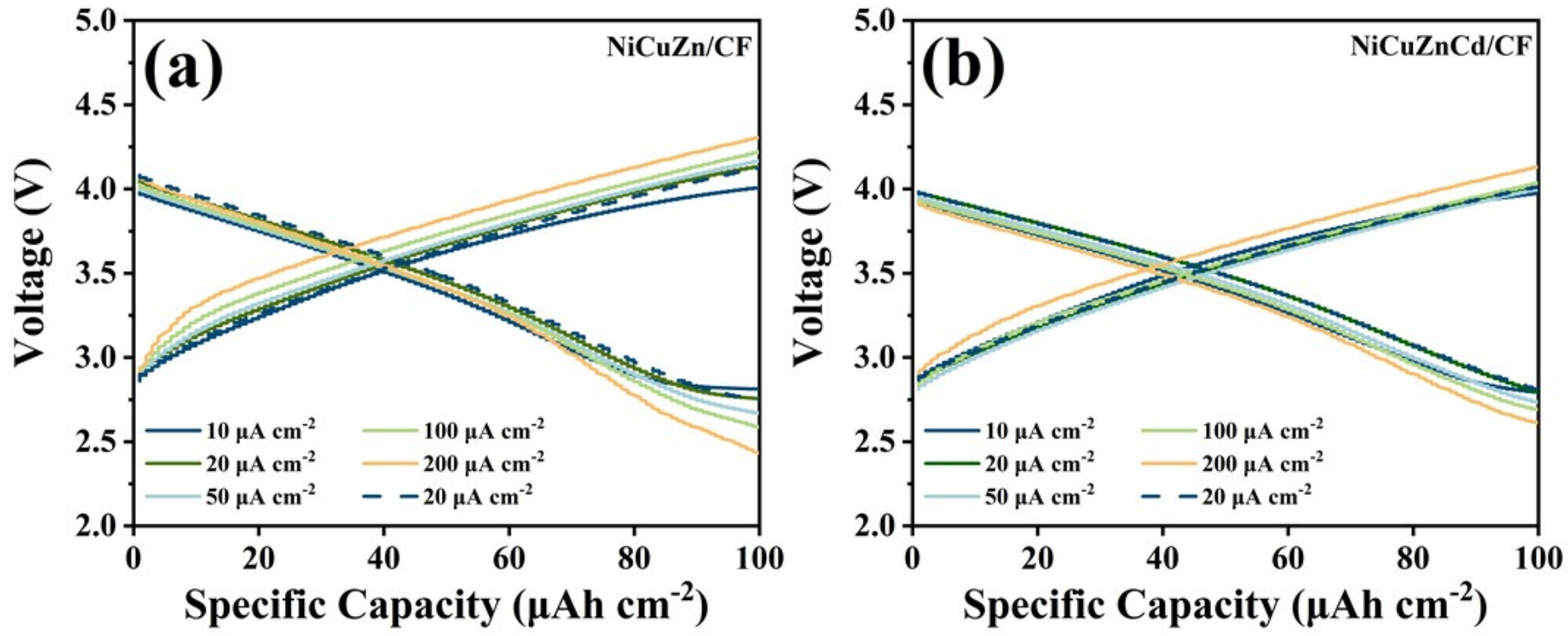

Fig. S9. GDC profiles of a limited capacity 100 μA h cm^{-2} at different current densities for (a) NiCuZn/CF and (b) NiCuZnCd/CF.

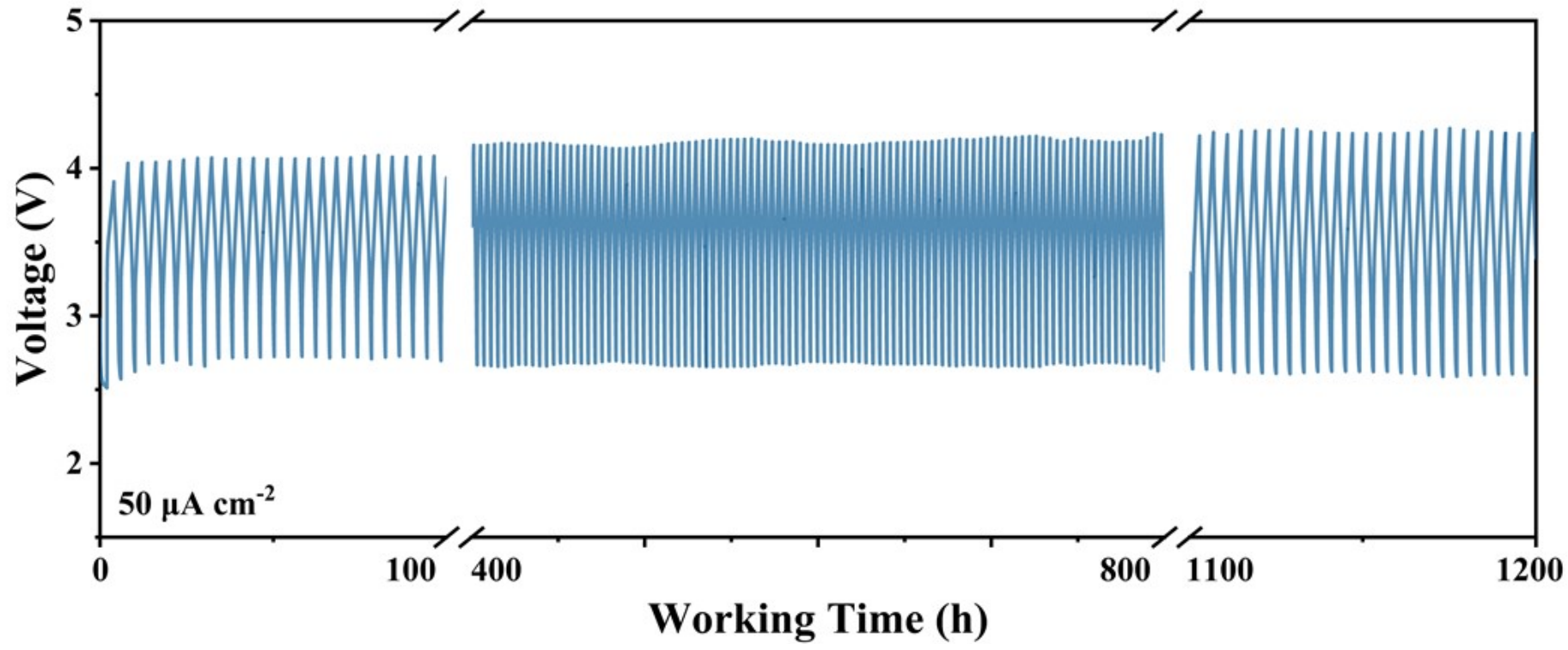

Fig. S10. Cyclability of NiCuZnCdRu/CF cathodes at 50 μA cm^{-2}.

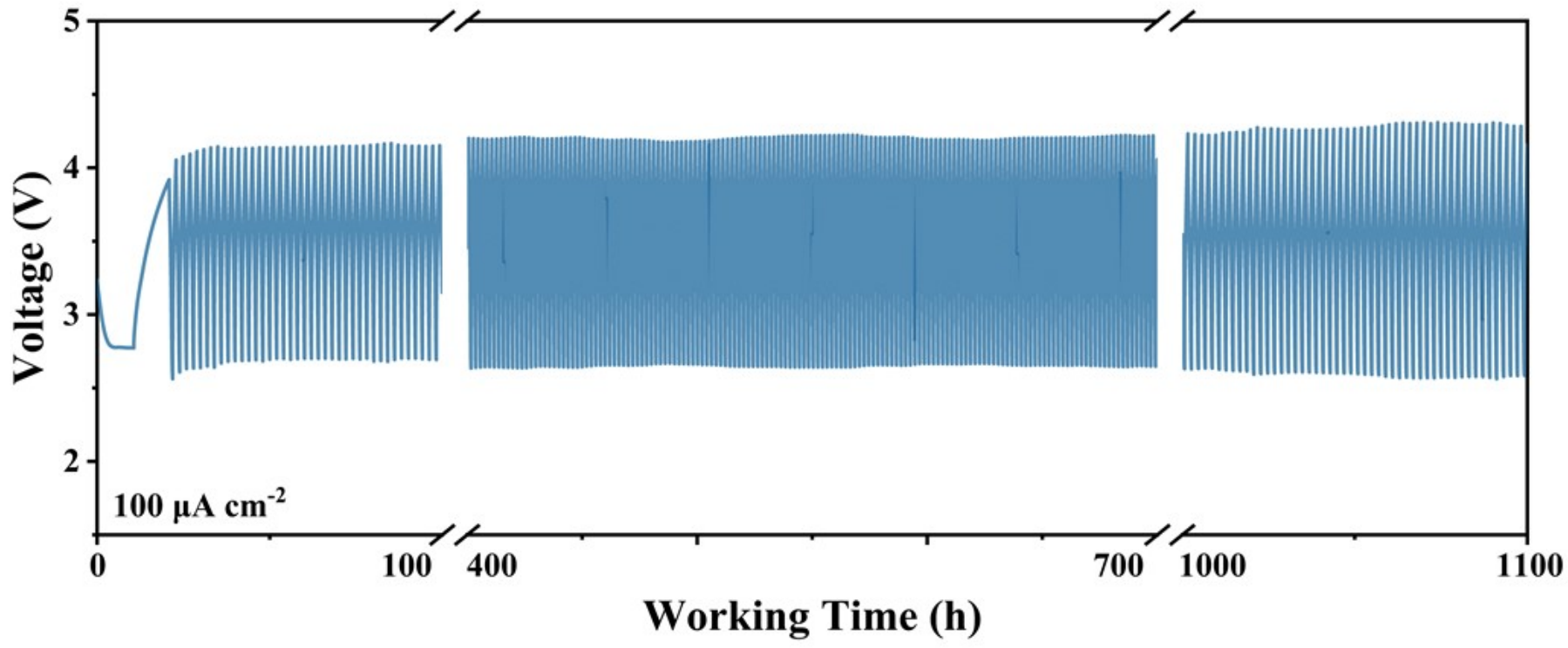

Fig. S11. Cyclability of NiCuZnCdRu/CF cathodes at 100 μA cm^{-2}.

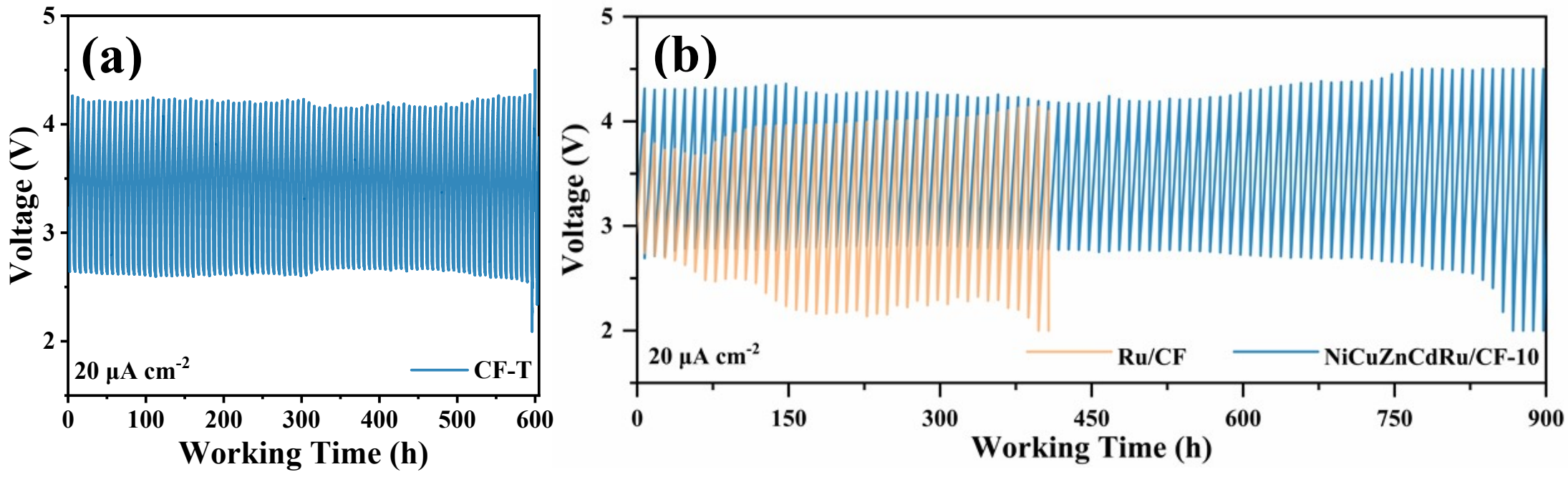

Fig. S12. Cyclability of (a) CF-T, (b) Ru/CF and NiCuZnCdRu/CF cathodes at 20 μA cm^{-2}.

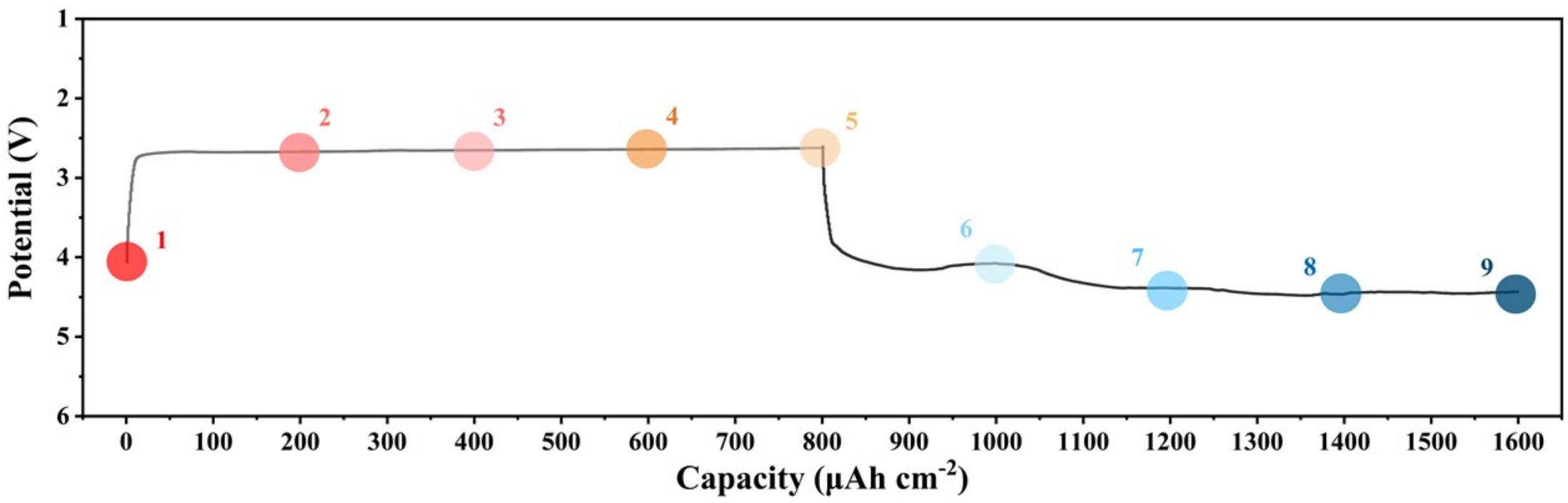

Fig. S13. GDC curve of the NiCuZnCdRu/CF cathode at 20 μA cm^{-2} with a capacity limit of 800 μAh cm^{-2}, showing the voltage profile at various states: (1) pristine, (2–5) discharge to 200, 400, 600, 800 μAh cm^{-2}, and (6–9) charge to 200, 400, 600, 800 μAh cm^{-2}.

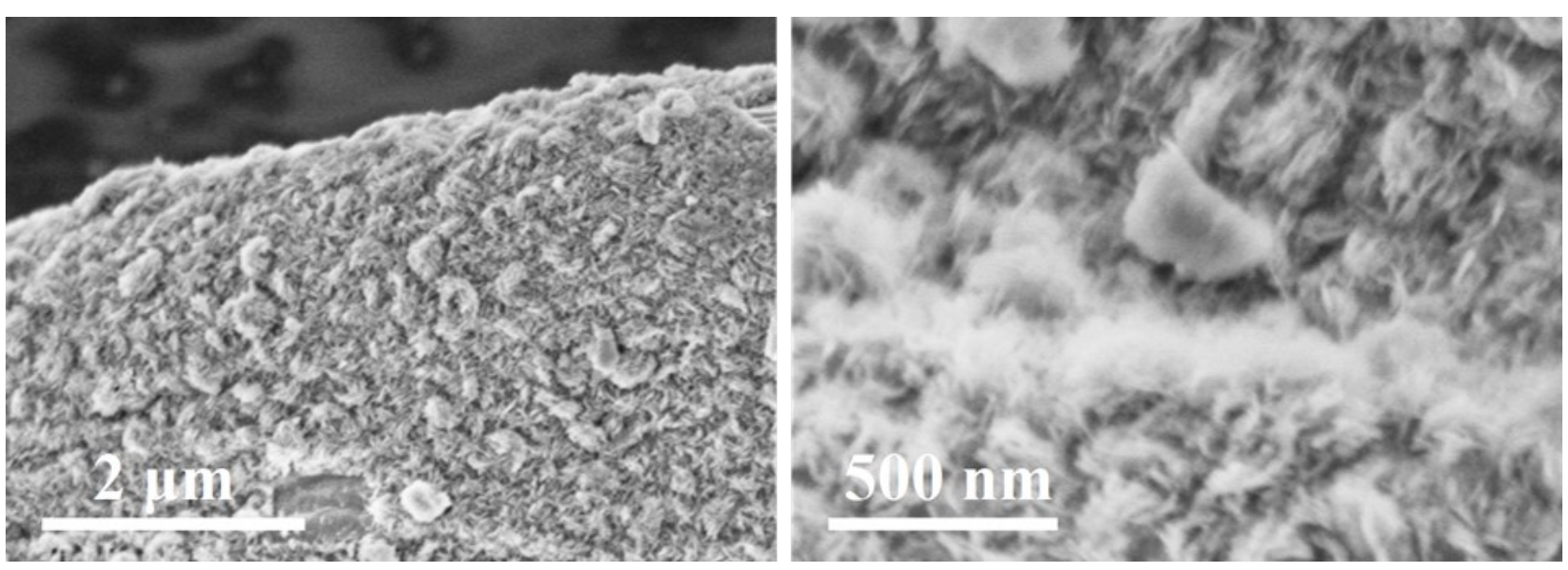

Fig. S14. SEM images of CF cathode at a limited discharge capacity of 400 μAh cm^{-2}.

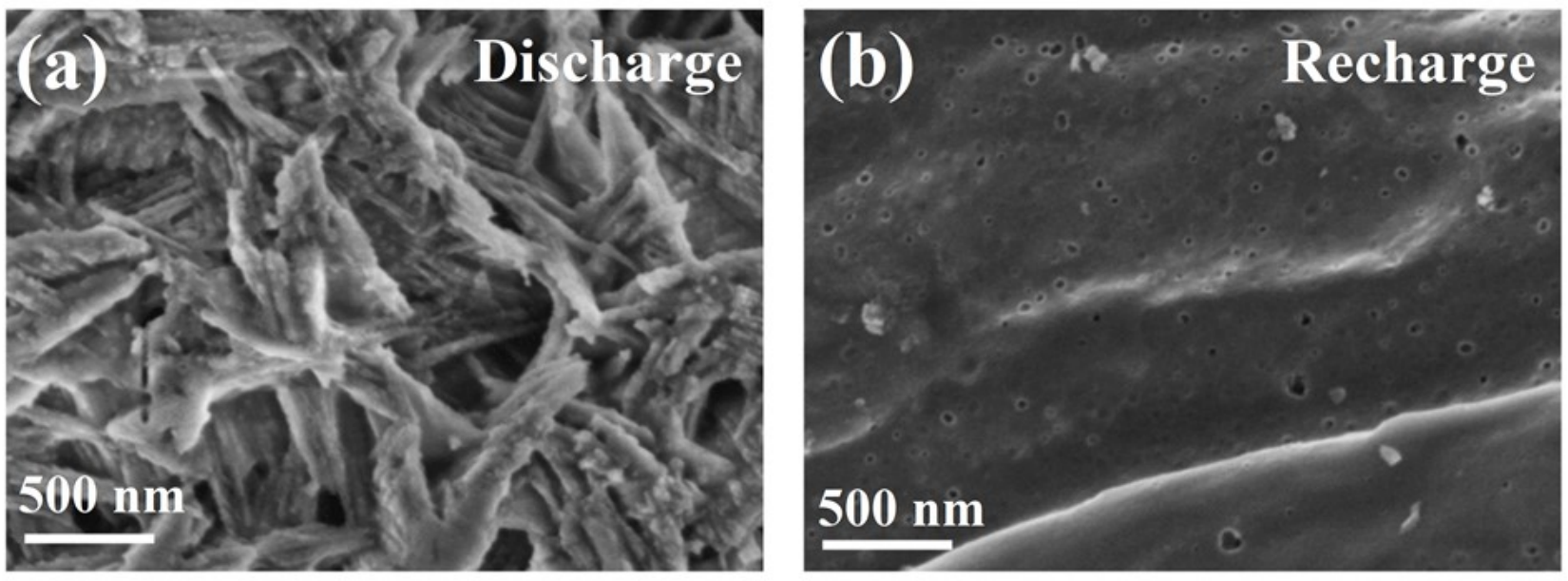

Fig. S15. SEM images NiCuZnCdRu/CF cathodes in different states.

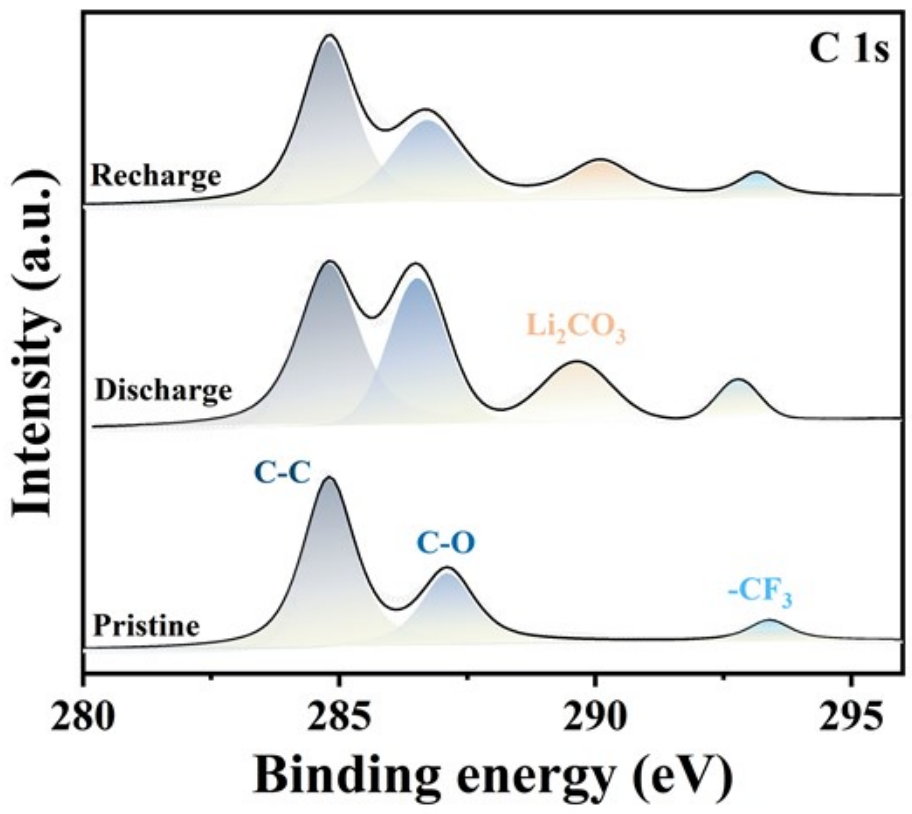

Fig. S16. C 1s XPS spectra of $Li-CO_2$ batteries with NiCuZnCdRu/CF cathode under different electrochemical stages, respectively.

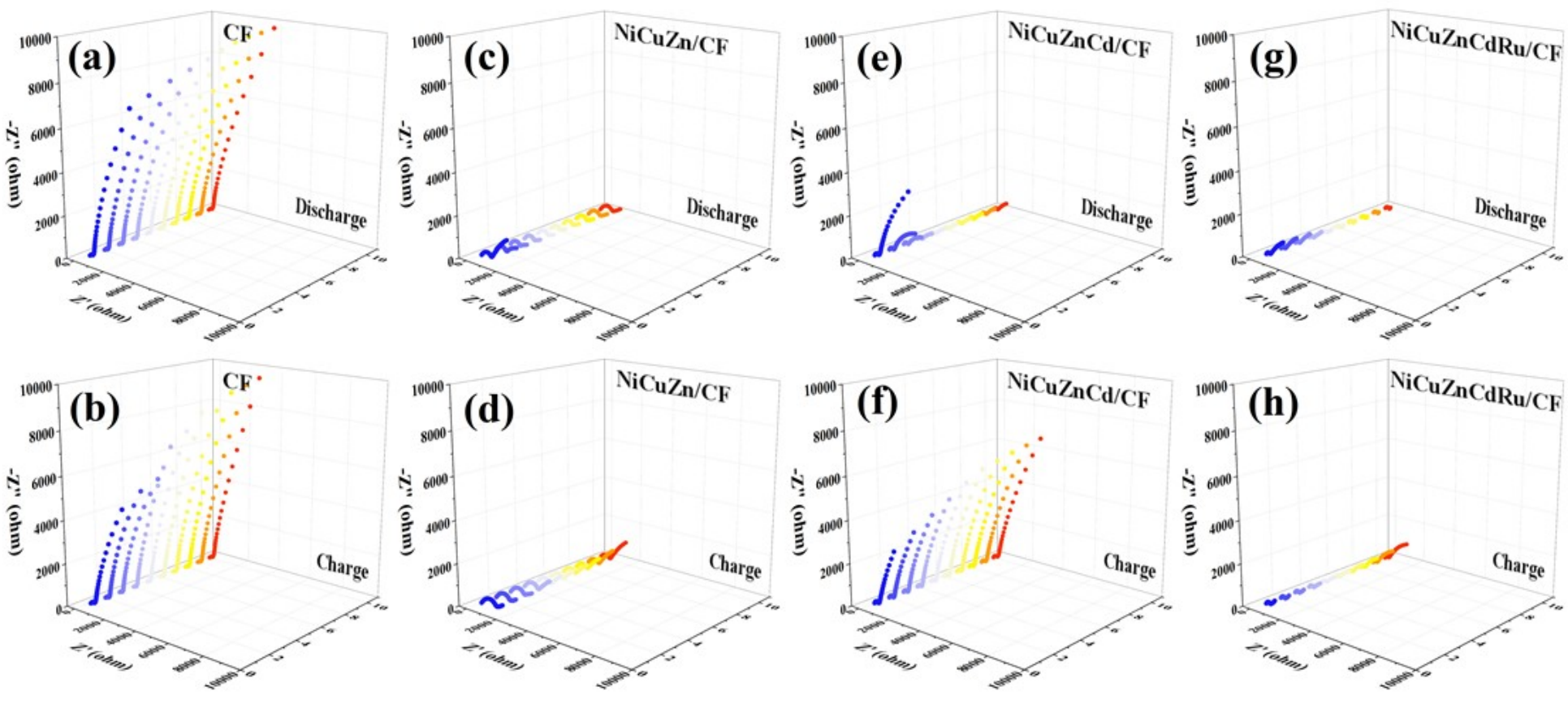

Fig. S17. In-situ EIS spectra of (a,b) CF , (c,d) NiCuZn/CF, (e,f) NiCuZnCd/CF, and (g,h) NiCuZnCdRu/CF cathodes discharge and charging processes.

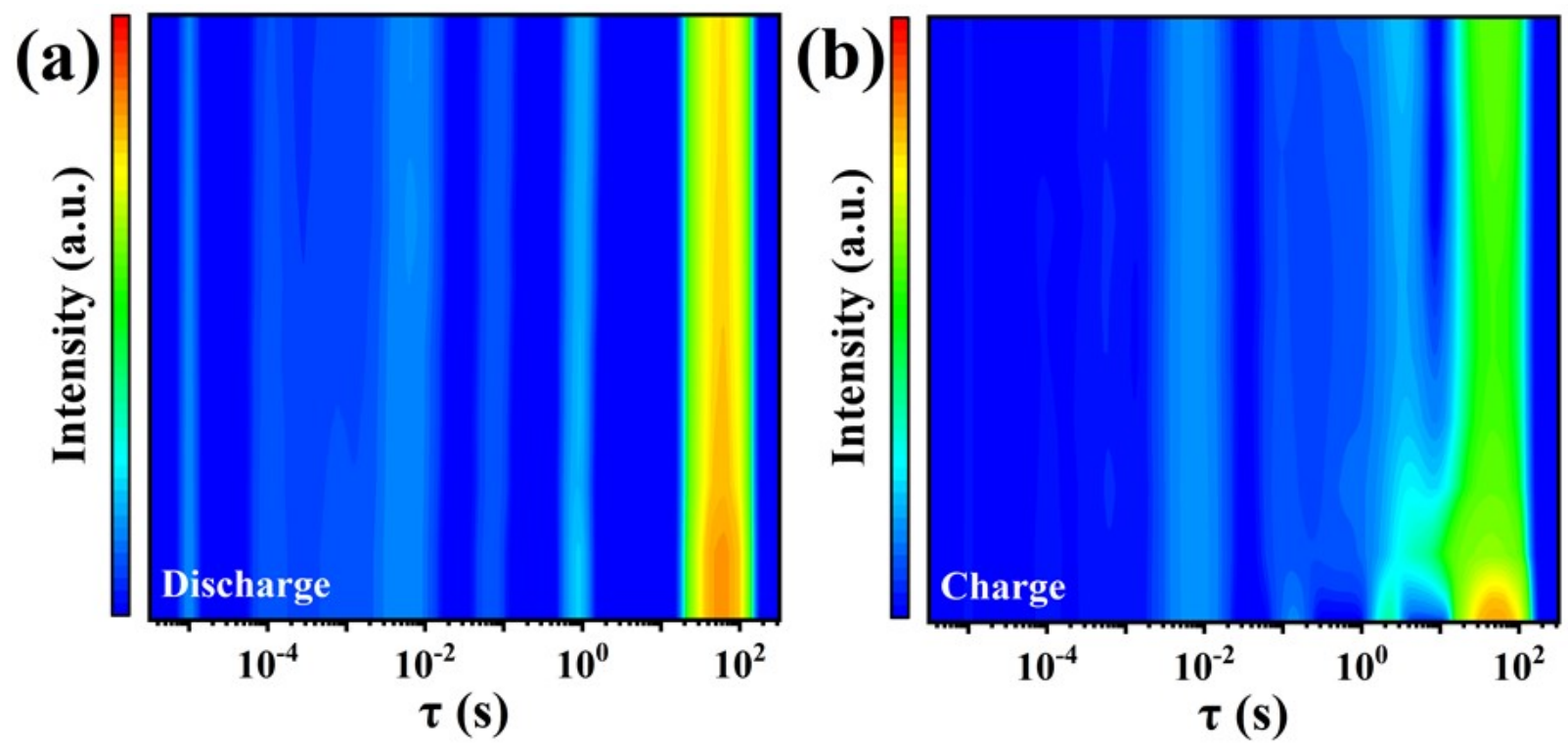

Fig. S18. 2D intensity color maps of the distribution of DRT for the Ru/CF cathodes.

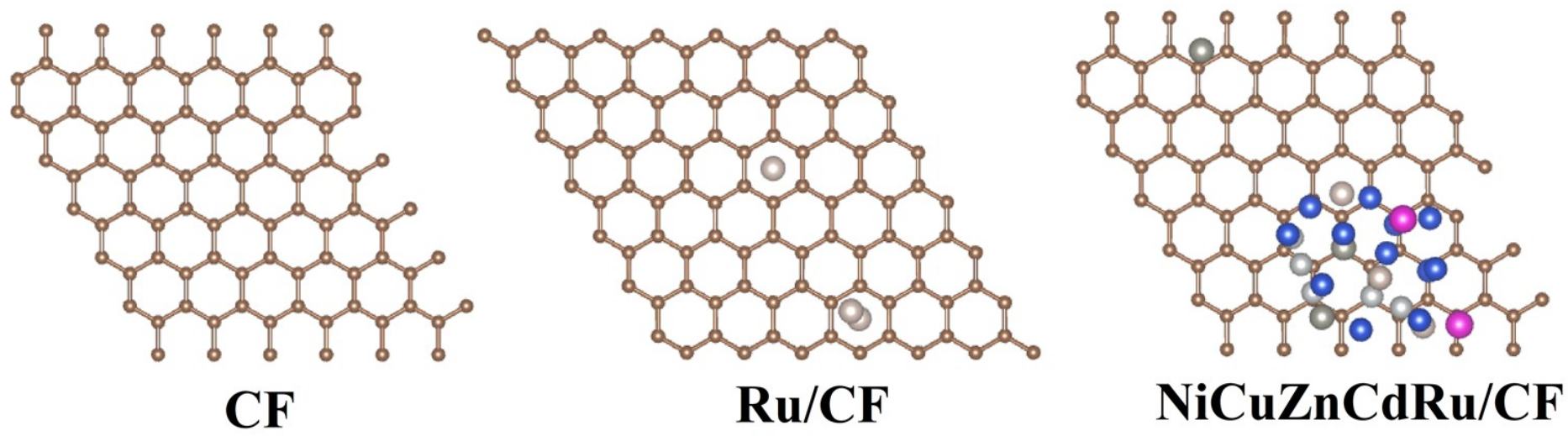

Fig. S19. The DFT model structures of CF, Ru/CF, and NiCuZnCdRu/CF.

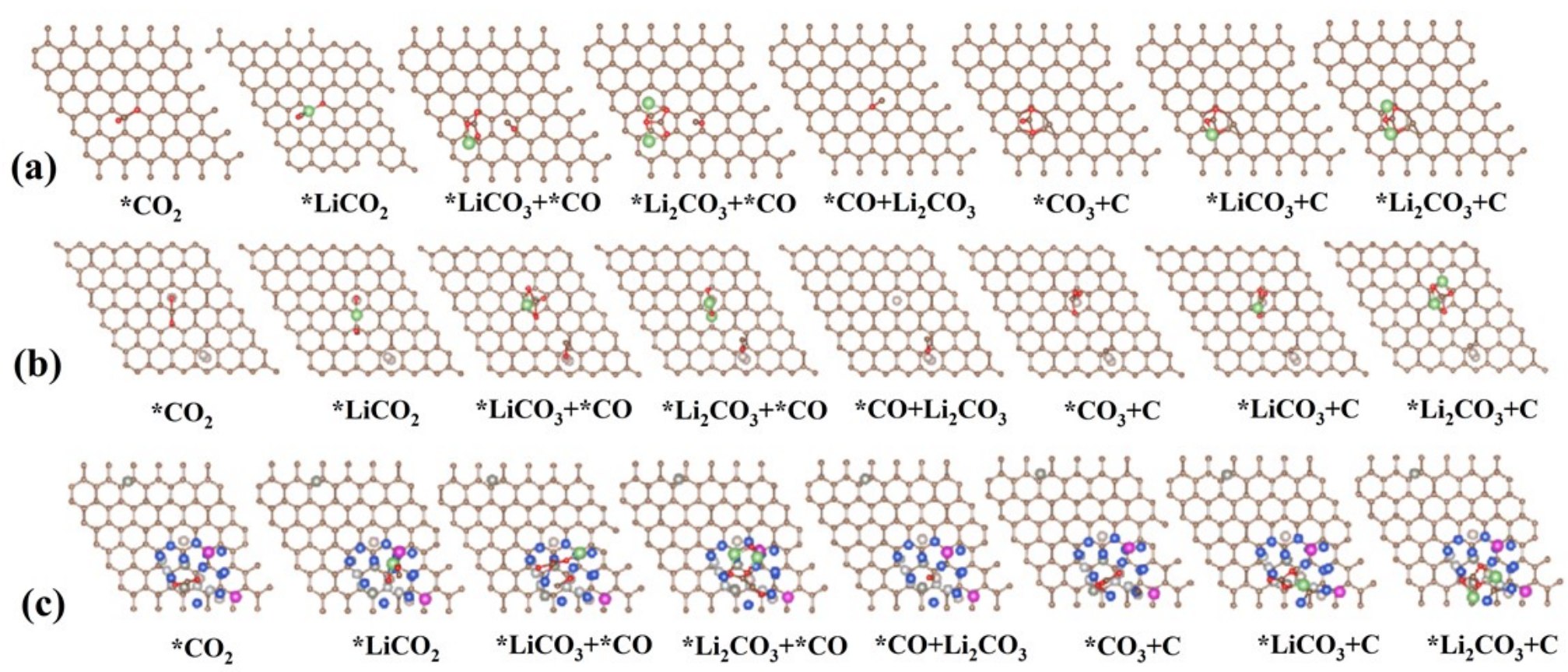

Fig. S20. The simulated configurations of intermediates during discharging processes of Li-CO_2 battery on (a) CF, (b) Ru/CF, and (c) NiCuZnCdRu/CF.

Fig. S21. Charge density differences plots key intermediates on NiCuZnCdRu/CF, yellow and cyan regions represent charge accumulation and depletion, respectively.

Fig. S22. SEM images of the cathodes of NiCuZnCdRu/CF battery after 2000 h cycling at 20 μA cm^{-2}.

Supplementary Tables

Table S1. ICP-MS composition of NiCuZn/CF, NiCuZnCd/CF and NiCuZnCdRu/CF.

Sample	Elements	Content(mg kg^{-1})	Wt.%
NiCuZnCdRu/CF	Ru	19.5	0.0019
	Cd	14.5	0.0014
	Cu	64.4	0.0064
	Ni	27.9	0.0028
	Zn	14.8	0.0015
NiCuZnCd/CF	Cd	1.4	0.0001
	Cu	56.4	0.0056
	Ni	32.1	0.0032
	Zn	19.2	0.0019
NiCuZn/CF	Cu	84.5	0.0085
	Ni	44.5	0.0045
	Zn	16.7	0.0017

Table S2. EXAFS fitting results for different materials at Zn K-edge.

Materials	pair	CN	R (Å)	σ^2 (Å^2)	ΔE (eV)	R factor
**Zn foil**	Zn-Zn	6(amp=0.77)	2.645 ± 0.031	0.0108	2.073	0.006
	Zn-Zn	6	2.790 ± 0.076	0.0229		
**ZnO**	Zn-O	4.0	1.959 ± 0.016	0.0018	2.846	0.020
	Zn-Zn	15.9	3.219 ± 0.005	0.0093		
**NiCuZnCdRu/CF**	Zn-C	6.8	1.901 ± 0.073	0.0011	-7.430	0.014
	Zn-M	3.5	2.623 ± 0.001	0.0054		

CN is the coordination number; Bond length (R(Å)) is the interactomic distance; σ^2 is Debye-Waller factor (a measure of thermal and static disorder in absorber scatter distance); ΔE is edge-energy shift (the difference between the zero kinetic energy value of the sample and that of the theoretical model); R factor is used to value the goodness of the fitting.

Table S3. Rate performance of NiCuZn/CF, NiCuZnCd/CF and NiCuZnCdRu/CF.

**Electrodes**	**Median discharge voltage /Median charge voltage**	**Voltage gap/Energy efficiency**	**Current densities ($\mu A\ cm^{-2}$)**
NiCuZnCdRu/CF	3.38, 3.48, 3.49, 3.48, 3.47 / 3.57, 3.58, 3.59, 3.61, 3.66	1.12, 1.19, 1.26, 1.28, 1.45 / 95.5%, 97.2%, 96.6%, 95.5%, 93.9%	10, 20, 50, 100, 200
NiCuZnCd/CF	3.40, 3.49, 3.44, 3.42, 3.37 / 3.60, 3.55, 3.53, 3.57, 3.66	1.11, 1.26, 1.32, 1.41, 1.65 / 95.2%, 98.0%, 97.4%, 95.5%, 91.5%	10, 20, 50, 100, 200
NiCuZn/CF	3.37, 3.45, 3.41, 3.41, 3.41 / 3.63, 3.67, 3.69, 3.74, 3.82	1.27, 1.38, 1.50, 1.64, 1.87 / 93.8%, 93.7%, 92.1%, 90.5%, 87.6%	10, 20, 50, 100, 200
CF	3.05, 3.05, 3.01, 2.95, 2.77 / 4.02, 3.94, 3.90, 3.92, 4.03	1.47, 1.56, 1.66, 1.88, 2.18 / 83.5%, 84.5%, 83.3%, 81.1%, 75.5%	10, 20, 50, 100, 200

Table S4. Comparison of the voltage gaps and energy efficiency for Li-CO_2 batteries.

**Cathode catalysts**	**Voltage gap**	**Energy efficiency**	**Current density**	**Reference**
Topological defect-rich graphene (TDG)	1.12	71.4%	100 mA g^{-1}	8
GNR/Ru	1.34	66.4%	100 mA g^{-1}	9
Ru-MHCS	1.17	71.5%	200 mA g^{-1}	10
PC/CoNi-CNTs	1.31	68.1%	20 μA cm^{-2}	11
NiO-Vo NAs/CT	1.3	66.8%	100 mA g^{-1}	12
MoS_2/CNT	1.22	68.5%	100 mA g^{-1}	13
NH_2-Cu-MOFs@RuO_2	1.06	73.1%	20 μA cm^{-2}	14
MnTPzP-Mn	1.41	65.6%	200 mA g^{-1}	15
Fe_1/N-rGO	1.45	65.6%	200 mA g^{-1}	16
SA Ru-Co_3O_4/CC	1.05	72.3%	100 mA g^{-1}	17
V-ACO/CC	0.77	93.1%	50 μA cm^{-2}	18
CoSe2/CMF	0.79	83.5%	20 μA cm^{-2}	19
CoS-Vs-1.0	0.62	82.8%	20 μA cm^{-2}	20
TVGA	1.41	66.3%	100 mA g^{-1}	21
**NiCuZnCdRu/CF**	**1.19**	**97.2%**	**20 μA cm^{-2}**	**This work**
	**1.26**	**96.6%**	**50 μA cm^{-2}**	

Table S5. Calculation results of DEMS data for NiCuZnCdRu/CF, NiCuZnCd/CF, and NiCuZn/CF.

	**NiCuZnCdRu/CF**	**NiCuZnCd/CF**	**NiCuZn/CF**
**CO_2 increase (measured value)**	5.9 μmol	5.4 μmol	3.2 μmol
**CO_2 increase (theoretical)**	7 μmol	7 μmol	7 μmol
**CO_2 conversion rate**	84.3%	77.7%	45.4%

The theoretical amount of CO_2 is calculated based on the assumption that all discharge products are formed exclusively via the 4-electron pathway ($4Li^+ + 3CO_2 + 4e^- \leftrightarrow 2Li_2CO_3 + C$), and on the cut-off charging capacity determined by the constant current charging duration.

Table S6. Bader charges of the metal atoms and adsorbates during the adsorption process.

Adsorption intermediate	Ni (e)	Cu (e)	Zn (e)	Cd (e)	Ru (e)	Adsorbate charge, Q_{ads} (e)
$*LiCO_2$	+0.0163	+0.0005	+0.2531	+0.0445	+0.1541	−0.106
$*LiCO_3$+*CO	+0.0260	+0.0575	+0.2255	+0.0404	+0.1677	−1.024
$*Li_2CO_3$+*CO	+0.0143	+0.0304	+0.2631	+0.3294	+0.0638	−0.915
$*CO_3$ + C	+0.0140	+0.0617	+0.2221	+0.2069	+0.1669	−1.352

Table S7. Comparison of the cyclic performance of NiCuZnCdRu/CF with previously reported.

Cathode catalysts	Voltage gap	Current density	Working Time	Reference
$NiCo_2S_4$	1.19 V	20 μA cm^{-2}	600 h	22
	1.45 V	100 μA cm^{-2}	250 h	
NGCA	1.22 V	20 μA cm^{-2}	1500 h	23
CFB@NCNTMo_2N	1.82 V	40 μA cm^{-2}	675	24
V-MoS_2/Co_9S_8@CP	1.04 V	20 μA cm^{-2}	630 h	25
Co_3O_4	1.00 V	20 μA cm^{-2}	600 h	26
	1.08 V	10 μA cm^{-2}	770 h	
Ex-$Ti_3C_2T_x$	0.94 V	100 μA cm^{-2}	306 h	27
$CoSe_2$/CMF	1.41 V	100 μA cm^{-2}	600 h	19
MOC@NCNF	1.45 V	20 μA cm^{-2}	1710 h	28
MoB	1.49 V	40 μA cm^{-2}	1400 h	29
	0.95 V	70 μA cm^{-2}	1400 h	
CP@NV-WN-500	1.27 V	20 μA cm^{-2}	600 h	30
	1.45 V	100 μA cm^{-2}	240 h	
V-ACO/CC	1.60V	100 μA cm^{-2}	420 h	18
**NiCuZnCdRu/CF**	**1.26 V**	**20 μA cm^{-2}**	**2500 h**	**This work**
	**1.56 V**	**50 μA cm^{-2}**	**1200 h**	
	**1.66 V**	**100 μA cm^{-2}**	**1100 h**	

References

1 P. Hohenberg and W. Kohn, *Phys. Rev.*, 1964, **136**, B864–B871.

2 W. Kohn and L. J. Sham, *Phys. Rev.*, 1965, **140**, A1133–A1138.

3 P. E. Blöchl, *Phys. Rev. B*, 1994, **50**, 17953–17979.

4 G. Kresse and J. Furthmüller, *Phys. Rev. B*, 1996, **54**, 11169–11186.

5 J. P. Perdew, K. Burke and M. Ernzerhof, *Phys. Rev. Lett.*, 1996, **77**, 3865–3868.

6 S. Grimme, J. Antony, S. Ehrlich and H. Krieg, *J. Chem. Phys.*, 2010, **132**, 154104.

7 S. L. Dudarev, G. A. Botton, S. Y. Savrasov, C. J. Humphreys and A. P. Sutton, *Phys. Rev. B*, 1998, **57**, 1505–1509.

8 F. Ye, L. Gong, Y. Long, S. N. Talapaneni, L. Zhang, Y. Xiao, D. Liu, C. Hu and L. Dai, *Advanced Energy Materials*, 2021, **11**, 2101390.

9 X. Ye, W. Liu, Y. Lu, X. Zheng, Y. Bi, M. Zheng, L. Han, B. Liu, Y. Ning, S. H. M. Jafri, X. Zhao, S. He, S. Zhang and H. Li, *J. Mater. Chem. A*, 2024, **12**, 10713–10725.

10 Y. Bai, L. Wei, Y. Lian, Z. Wei, D. Song, Y. Su, X. Zhu, W. Huo, J. Cheng, Y. Peng and Z. Deng, *ACS Appl. Mater. Interfaces*, 2023, **15**, 41457–41465.

11 X. Ji, Y. Liu, Z. Zhang, J. Cui, Y. Fan and Y. Qiao, *Journal of Colloid and Interface Science*, 2024, **655**, 693–698.

12 C. Wang, Y. Lu, S. Lu, S. Ma, X. Zhu, Z. Li and Q. Liu, *Journal of Power Sources*, 2021, **495**, 229782.

13 C.-J. Chen, C.-S. Huang, Y.-C. Huang, F.-M. Wang, X.-C. Wang, C.-C. Wu, W.-S. Chang, C.-L. Dong, L.-C. Yin and R.-S. Liu, *ACS Appl. Mater. Interfaces*, 2021, **13**, 6156–6167.

14 H. Hong, J. He, Y. Wang, X. Guo, X. Zhao, X. Wang, C. Zhi, H. Li and C. Han, *J. Mater. Chem. A*, 2022, **10**, 18396–18407.

15 L.-Z. Dong, Y. Zhang, Y.-F. Lu, L. Zhang, X. Huang, J.-H. Wang, J. Liu, S.-L. Li and Y.-Q. Lan, *Chem. Commun.*, 2021, **57**, 8937–8940.

16 L. Zhou, H. Wang, K. Zhang, Y. Qi, C. Shen, T. Jin and K. Xie, *Sci. China Mater.*, 2021, **64**, 2139–2147.

17 Z. Lian, Y. Lu, C. Wang, X. Zhu, S. Ma, Z. Li, Q. Liu and S. Zang, *Advanced Science*, 2021, **8**, 2102550.

18 J. Yang, Y. Liu, Y. Liu, Y. Huang, K. Wang, M. Liu and W. Li, *Nano Energy*, 2024, **129**, 109979.

19 K. Wang, L. Liu, D. Liu, Y. Wei, Y. Liu, X. Wang, A. S. Vasenko, M. Li, S. Ding, C. Xiao and H. Pan, *Small*, 2024, **20**, 2310530.

20 B. Lu, X. Wu, M. Zhang, X. Xiao, B. Chen, Y. Liu, R. Mao, Y. Song, X.-X. Zeng, J. Yang and G. Zhou, *J. Am. Chem. Soc.*, 2024, **146**, 20814–20822.

21 W. Zhao, Y. Yang, Q. Deng, Q. Dai, Z. Fang, X. Fu, W. Yan, L. Wu and Y. Zhou, *Advanced Functional Materials*, 2023, **33**, 2210037.

22 Y. Chen, J. Li, B. Lu, Y. Liu, R. Mao, Y. Song, H. Li, X. Yu, Y. Gao, Q. Peng, X. Qi and G. Zhou, *Advanced Materials*, 2024, **36**, 2406856.

23 W. Yu, L. Liu, Y. Yang, N. Li, Y. Chen, X. Yin, J. Niu, J. Wang and S. Ding, *Chemical Engineering Journal*, 2023, **465**, 142787.

24 L. Chen, J. Zhou, Y. Wang, Y. Xiong, J. Zhang, G. Qi, J. Cheng and B. Wang, *Advanced Energy Materials*, 2023, **13**, 2202933.

25 B. Lu, B. Chen, D. Wang, C. Li, R. Gao, Y. Liu, R. Mao, J. Yang and G. Zhou, *Proceedings of*

the National Academy of Sciences, 2023, **120**, e2216933120.
26 Y. Liu, P. Shu, M. Zhang, B. Chen, Y. Song, B. Lu, R. Mao, Q. Peng, G. Zhou and H.-M. Cheng, *ACS Energy Lett.*, 2024, **9**, 2173–2181.
27 X. Li, X. Wang, M. Yang, H. Meng, J. Yuan, Q. Yi, Z. Cao, K. Hou, K. Qi, L. Gao, J. Cheng, B. Wang and J. Wang, *Advanced Materials*, 2025, **37**, 2500064.
28 L. Liu, S. Shen, N. Zhao, H. Zhao, K. Wang, X. Cui, B. Wen, J. Wang, C. Xiao, X. Hu, Y. Su and S. Ding, *Advanced Materials*, 2024, **36**, 2403229.
29 Y. Liu, T. Liu, X. Wang, J. Zhang, X. Zhai, T. Wei, Q. Shi, C. Lu, H. Yan, Y. Xia, W. Cheng and M. Zhou, *Advanced Materials*, 2025, **n/a**, 2507871.
30 H. Xie, Y. Zhang, B. Chen, C. He, C. Shi, E. Liu and N. Zhao, *Journal of Energy Chemistry*, 2024, **97**, 585–592.
